# The Chicken Embryo Model: A Novel and Relevant Model for Immune-Based Studies

**DOI:** 10.3389/fimmu.2021.791081

**Published:** 2021-11-19

**Authors:** Paul Garcia, Yan Wang, Jean Viallet, Zuzana Macek Jilkova

**Affiliations:** ^1^ Université Grenoble Alpes, Grenoble, France; ^2^ R&D Department, Inovotion, La Tronche, France; ^3^ Institute for Advanced Biosciences, Research Center Université Grenoble Alpes (UGA)/Inserm U 1209/CNRS 5309, La Tronche, France; ^4^ Service d’Hépato-Gastroentérologie, Pôle Digidune, Centre Hospitalo-Universitaire (USA) Grenoble Alpes, La Tronche, France

**Keywords:** immunology, chicken, chick embryo, chicken embryo, preclinical model, chorioallantoic membrane, ontogeny, egg

## Abstract

Dysregulation of the immune system is associated with many pathologies, including cardiovascular diseases, diabetes, and cancer. To date, the most commonly used models in biomedical research are rodents, and despite the various advantages they offer, their use also raises numerous drawbacks. Recently, another *in vivo* model, the chicken embryo and its chorioallantoic membrane, has re-emerged for various applications. This model has many benefits compared to other classical models, as it is cost-effective, time-efficient, and easier to use. In this review, we explain how the chicken embryo can be used as a model for immune-based studies, as it gradually develops an embryonic immune system, yet which is functionally similar to humans’. We mainly aim to describe the avian immune system, highlighting the differences and similarities with the human immune system, including the repertoire of lymphoid tissues, immune cells, and other key features. We also describe the general *in ovo* immune ontogeny. In conclusion, we expect that this review will help future studies better tailor their use of the chicken embryo model for testing specific experimental hypotheses or performing preclinical testing.

## 1 Introduction

The immune system has vital functions for the organism, from defense to homeostasis. Its efficiency is mainly based on a constant balance between activation and tolerance, which helps protect the organism against dangers such as pathogens and abnormal cells, without disturbing its own cells. However, this balance can sometimes be fragile and might eventually be disturbed, leading to immune-related diseases. These pathologies are diverse: besides immune deficiency and auto-immune diseases, these also include less-obvious ones, such as cancer, diabetes, and many cardiovascular conditions (1). The high prevalence of these diseases underlines the need for therapeutic solutions for patients suffering from these pathologies (2–4). Therefore, it is now essential to conduct immunology-based studies to better understand the pathways involved, but also to develop new treatments.

The immune system is very complex and involves the whole organism, with many cells, cytokines, chemokines, and other proteins interacting together, which cannot be fully replicated *in vitro*. Currently, the most widely used animals in biomedical research are rodents. As mammals, their biological functions, including the immune system, are well-described and comparable to humans. Despite many advantages, rodent models are far from optimal. First, there are obvious ethical issues that are raised with the use of laboratory animals, with more than 100 million rodents believed to be used for experiments each year in the U.S. alone (5). Many biological experiments are potentially painful for animals, including immune system-related experiments. Indeed, disturbing the rodent immune system can exhibit a great impact on the animal’s well-being. For instance, injecting lipopolysaccharide (LPS), one of the most used modulators to induce inflammation, can have a dramatic impact on mice, with the appearance of depressive-like behaviors (6) and neurological damage (7). Besides the obvious ethical issues, there are also substantial differences between the rodent and human immune systems (8).

Although rodents represent a pertinent model for biomedical research, other models are also very promising. One well-established *in vivo* model that is gaining increased interest is the chicken embryo model, and more importantly, the use of its chorioallantoic membrane (CAM) (9). Indeed, the avian CAM functions as a homolog of the mammalian placenta, which provides the egg with a rich capillary vascular network and an interface for gas exchange (10, 11). It can easily be used in biomedical research for a multitude of applications, such as evaluating angiogenesis, tumor growth, metastasis, and therapy responses (12–18). Furthermore, this model is far from new: Rous and Murphy initially used it in 1911 to demonstrate the growth of chicken sarcoma tumors transplanted onto the CAM (19, 20). It has since been extensively studied and described, and it is very well understood today. This review will therefore attempt to describe and explain the various advantages of the chicken embryo model, and its relevancy for immune-based studies.

## 2 Overview of the Mature Chicken Immune System

**Figure 1:**
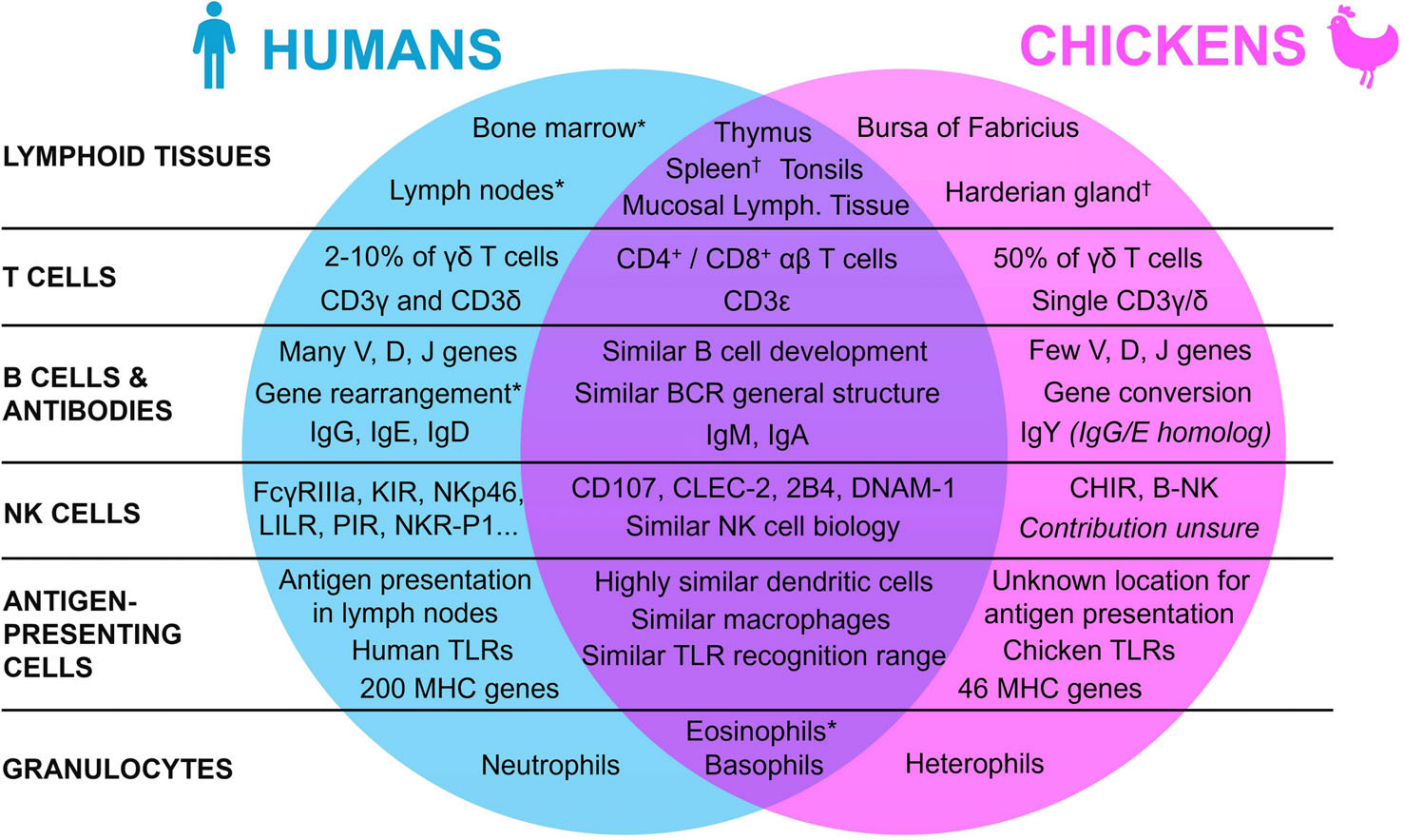
Summary of the similarities and differences between the human and the chicken immune systems. * Present in both species, with a lower contribution in chickens. † Present in both species, with a lower contribution in humans.

To understand the use of the chicken embryo model, it is essential to first understand the mature avian immune system, by highlighting the similarities and differences with the human immune system. Indeed, since the avian organism shows many specific differences with the mammal, either in its repertoire of lymphoid tissues, immune cells, and molecules, this review will first broaden its scope to encompass general lymphoid tissue morphology, before focusing on the various components of the immune system itself (**Figure 1**).

### 2.1 Morphology of the Lymphoid System

Lymphoid organs are an essential aspect of the immune system of all vertebrates. They can be divided into two categories based on their functions: the primary and the secondary lymphoid organs. Even though these organs appeared early in the evolutionary tree with their functions mainly conserved, they may differ between species (21–23). In humans, primary lymphoid tissues are composed of the thymus and the bone marrow. They host the development and maturation of hematopoietic progenitors, which can differentiate into a wide panel of immunologically competent cells depending on the organ (24, 25). In both humans and chickens, the thymus is located around the pharynx and is mainly composed of reticular epithelial cells, macrophages, and fibroblasts that are all involved in the formation of the T cell repertoire (26–28). While the thymus functions and location are quite similar in both species with slight differences in morphology (*e.g.*, humans have 2 lobes whereas chickens have 7-8 lobes), the organ for B cell maturation is however entirely different (29). Indeed, in chickens, this maturation doesn’t occur in the bone marrow, but in an organ specific to birds: the bursa of Fabricius, a diverticulum located near the cloaca, which is also the site of development of the antibody repertoire (30). The “B” in B cells actually refers to this bursa, reflecting the historical origins of B cell discovery (31, 32). The follicles of the bursa are composed of reticular epithelial cells, bursal secretory dendritic cells, mature T helper cells, macrophages, and B cells at different stages of maturation (33). In this organ, most of the lymphoid cells are B cells (98%), with only 2% of T cells (27). Even though the tissue for B cell generation differs between the two species, hematopoiesis in chickens, and mostly the generation of immune cell precursors, still occurs in the bone marrow as in humans (34, 35).

While primary lymphoid organs act mainly as the center of the adaptive immune cells’ generation and maturation, secondary lymphoid tissues instead specialize in the coordination of immune responses, namely by activating immune effector cells such as lymphocytes (22). After maturation in the primary lymphoid organs, T and B cells re-enter the bloodstream and colonize the secondary lymphoid tissues, to facilitate antigen presentation to lymphoid cells and initiate and regulate the adaptive immune response. In humans, the main secondary lymphoid tissues are the spleen, the tonsils, the lymph nodes, and the mucosa-associated lymphoid tissues. Even though they vary slightly in functionality and morphology, they all share the same basic structure, with a T cell zone and B cell follicles (36, 37). In mature chickens, the secondary lymphoid tissues are similar to those in humans, from their basic structural aspect to the organs themselves, but some differences must still be mentioned (29, 38). Indeed, even though there are also some slight differences in the organs’ morphology and location, the most striking distinction is that, unlike mammals, birds lack encapsulated lymph nodes. To compensate, other lymphoid tissues, namely the spleen and the Harderian gland, have a higher contribution to the chicken immune system (29, 39). Moreover, chickens have extensive mucosa-associated lymphoid tissues (*e.g.*, Peyer’s patches and Meckel’s diverticulum). While chickens do not have the classical lymphoid nodes, they do have instead rudimentary mural lymphoid nodules that are different both anatomically and histologically when compared to mammalian lymph nodes, with a lower contribution to the chicken immune system overall (29).

### 2.2 The Diverse Panel of the Avian Immune Cells

To build a proper immune response, the chicken, like all vertebrates, uses a wide panel of immune cells. Their respective biological functions, but also their coordination with each other through various mediators, allow them to contribute towards a robust defense against pathogens. Succinctly, the immune response can be subdivided into two categories: innate and adaptive immunity. In a matter of minutes, innate immunity employs a wide range of cells, such as dendritic cells, macrophages, granulocytes, and Natural Killer (NK) cells, to generate an inflammatory reaction and destroy infected cells and pathogens non-specifically. Adaptive immunity slowly develops within several days and involves T and B cells in an antigen-specific manner, as they are activated by antigen-presenting cells, *i.e.*, dendritic cells and macrophages, involved in innate immunity. This activation leads to the generation of antigen-specific antibodies and T helpers cells, allowing the vertebrate animal to build an efficient and complete defense against a given pathogen (40). To properly understand the chicken immune system, it is essential to describe the main cell populations involved in the immune response.

#### 2.2.1 Dendritic Cells

Dendritic cells (DCs) are the main antigen-presenting cells, capable of endocytosis, exocytosis, and cytokine production. In humans, DCs are composed of two major populations: conventional DCs (cDCs), previously called myeloid dendritic cells, which are further subdivided into two major subpopulations, cDC1 and cDC2, and plasmacytoid DCs (pDCs). cDCs are the professional antigen-presenting cells of the innate immune system. They reside in tissues, and after tissue infection or injury, they become activated and migrate to lymph nodes to promote adaptive immune responses (41). pDCs are distinct from cDCs in terms of their development and function. They arise from the lymphoid progenitor and their main function in the human immune response is type I and type III interferon secretion upon acute or chronic viral infection (42).

In both chickens and humans, DCs act as a bridge between the innate and the adaptive immune responses, though many differences are described in these heterogeneous cell types. For instance, a specific subset of DCs, present in birds but not in mammals, exist: the bursal secretory DCs, located in the bursa of Fabricius (43). Overall, chicken DCs have high plasticity, and all of these cell subsets share structural and functional differences with humans, from regulating central tolerance to shaping the adaptive immune response (44). A comprehensive review previously summarized the ontogeny, cytoarchitecture, and immunophenotype of avian DCs (45). To this day, knowledge of avian DCs still appears to be limited compared to that of mammals. Nevertheless, many aspects of mature DCs, including their morphology, their surface receptors, their response to stimuli, and their respective functions, seem to be highly similar between chickens and humans (45–47).

Chicken cDCs are the most thoroughly studied dendritic cell subset. Indeed, comparative gene expression profiling has been performed in chickens and humans, which demonstrated that cDC subsets are homologous (48, 49). A recent study also revealed the enrichment of cDC lineage in the chicken spleen (50). Still, a key aspect should be further investigated: the location of antigen presentation to lymphocytes. Indeed, while this phenomenon generally appears in vertebrates’ secondary lymphoid organs, it occurs mostly in lymph nodes in humans, which chickens lack as mentioned earlier. This location is still not properly identified in chickens yet, although it is hypothesized that lymphoid nodules, and several novel lymphoid structures and aggregates, might be involved in antigen presentation (46).

#### 2.2.2 Macrophages

Macrophages are an essential component of the innate immune system. Like dendritic cells, macrophages can present antigens to T cells. Their main function is to phagocytose cellular debris, pathogens, and damaged cells, but they also play a crucial role in tissue homeostasis and the host immune response by mediating inflammation (51). Under normal physiological conditions or during inflammation, circulating monocytes, the precursors of macrophages, migrate into an appropriate location according to chemotactic signals. After cytokine-mediated differentiation, mature macrophages are then capable of destroying a pathogen, producing cytokines capable of modulating other inflammatory cells, and secreting various growth factors to promote angiogenesis and tissue repair (52). Depending on the various mediators and cytokines present in their environment, immature macrophages from humans and chickens can be differentiated schematically into two main types of macrophages: M1 and M2. However, macrophages being extremely plastic, it is not possible to simply divide them into two phenotypes but are rather characterized by a range of intermediate phenotypes between the extremes (53, 54). In both species, while M1 macrophages stimulate inflammation, mainly by secreting IL-1β, TNF-α, and IL-6, M2 macrophages instead stimulate cell reparation and angiogenesis through secretion of IL-10, TGF-β, and VEGF (55–58).

Similar to humans, functional studies on chicken macrophages indicate the presence of scavenger receptors, complement receptors, Fc receptors, C-type lectins, and mannose receptors, all mediating the recognition of antigens before phagocytosis (59–62). Moreover, the production and release of nitric oxide, an important microbicidal mechanism of activated macrophages in humans, has been described in chicken macrophages with high homology (63–65). Finally, as in humans, IFN-γ can induce polarization of immature chicken macrophages into the pro-inflammatory M1 phenotype, while IL-4 can induce it into the anti-inflammatory M2 phenotype (53, 54, 66). However, although the mammalian peritoneal cavity contains non-activated resting macrophages, these cells are mainly absent in chickens. Instead, avian macrophages must be recruited from the bloodstream into the cavity by inflammatory stimuli (46). Interestingly, a recent study characterized several different macrophage subsets in the chicken spleen that may play an important role in antigen presentation and immune responses (50).

#### 2.2.3 Granulocytes

Granulocytes are described as non-specific immune cells containing granules (with defensins, lysozymes, histamines, *etc.*) in their cytoplasm. They are derived from common myeloid progenitors in the bone marrow, and they also contribute to the initial innate immune response against pathogens. In mammals, they can be divided into three subsets, with structural and functional differences: neutrophils, eosinophils, and basophils (67). Neutrophils are highly heterogeneous and the most abundant type of myeloid cells (68, 69), representing 50-70% of the total circulating human white blood cells. Neutrophils are released into the bloodstream with as a main function to phagocytose infected cells and destroy pathogens through degranulation (70). Eosinophils, however, represent a small number of circulating leukocytes (1-6%) and are mainly involved in fighting parasitic infections, but also in allergic diseases (71). Finally, basophils, accounting for less than 1% of total blood leukocytes, can release histamine and other mediators, which contribute to the inflammatory response (72). All these cells’ subsets migrate to peripheral and lymphoid tissues during inflammation.

Even though granulocytes represent essential cells of the chicken immune system, they do differ from mammals. Indeed, chickens lack neutrophils, but they do have a functional counterpart called heterophils. These cells are highly phagocytic as well, but in contrast to mammalian neutrophils they lack myeloperoxidase, they have a lower bactericidal activity through oxidative burst, and their granule components seem to differ (73). It is however still unknown whether heterophils represent a single set of cells with identical functions, or whether they encompass functionally different subsets of cells (74). Despite these differences, the biological functions of avian heterophils seem to be highly similar to mammalian neutrophils. They are the first cell type recruited to the inflammation site in response to chemokines. They express pattern recognition receptors (mostly Toll-like receptors) at their surface to recognize antigens, and they can eliminate pathogens through oxidative burst, degranulation, and extracellular traps (47, 75–77).

Even though eosinophils are present in chickens, they have yet to be demonstrated to be functional. For instance, IL-5 expression and IgE production, both essential for the response and function of mammalian eosinophils, are absent in chickens. Furthermore, eotaxins (CCL11, CCL24, and CCL26) and their receptor (CCR3), which are vital for eosinophils migration in mammals, are absent in the chicken genome (27, 78). Earlier studies also showed that avian eosinophils are less responsive to antigenic stimulation than in mammals (79). Basophils are present in chickens as well, and appear to have a function similar to their mammalian counterpart, with the induction of a hypersensitivity reaction through histamine release (80, 81).

#### 2.2.4 Natural Killer (NK) Cells

Natural Killer (NK) cells represent a lymphoid lineage that shares many features with cytotoxic T lymphocytes. Indeed, they are both defined as large granular lymphocytes, and they play an essential role in the innate immune response by eliminating infected and damaged cells. Even though NK cells are derived from a common lymphoid progenitor, their development is independent of the thymus in both species and, for chickens, of the bursa of Fabricius as well. For both mammals and chickens, the maturation process instead relies on the bone marrow (73, 82). NK cells’ functions rely mainly on a combination of their inhibitory and activating receptors (83, 84), which is a similar system in humans and chickens. Several homologs exist between the two species, namely CD107, CLEC-2, 2B4, and DNAM-1 (84–87). Furthermore, human NK cells can express KIR, NKp46, LILR, and PIR, whereas chickens cannot. They instead express the chicken Ig-like receptor (CHIR) family that may be used as a functional homolog of these receptors (27, 88). Likewise, chickens do not express NKR-P1 but have a functional equivalent, B-NK (87, 89). This suggests that chicken NK cell biology is close to mammalians’, although some other mammalian receptors do not seem to be expressed in chickens, and orthologs have yet to be found.

NK cells bear three main roles in both humans and chickens, although the underlying mechanisms can be slightly different. The first is a direct cytolytic activity, mainly through the secretion of perforin and granzyme, and surface expression of Fas ligand and TRAIL (90, 91). Another essential role of NK cells in both species is antibody-dependent cell-mediated cytotoxicity (ADCC), a mechanism activated by cross-linking of an activating Fc receptor with the Fc region of an antigen-bound antibody, leading to secretion of perforin, granzymes, and cytokines (92, 93). In humans, the activating Fc receptor mainly expressed by NK cells is FcγRIIIa (CD16a), a receptor specific for IgG (94). However, as described earlier, chickens are not able to produce IgG, and therefore do not express FcγRIIIa. To perform ADCC, they instead use another receptor, CHIR-AB1, a high-affinity Fc receptor for IgY (88, 95). Finally, the third role of NK cells is cytokine secretion, and more importantly of IFN-γ, involved in inflammation and macrophage activation in both species (96, 97).

While NK cell biological functions appear to be highly similar between humans and chickens, it is still unsure how much these cells contribute to the chicken immune response. Indeed, earlier studies showed that the frequency of NK cells in blood, spleen, and cecal tonsils was remarkably low (0.5 to 1% of blood lymphocytes) (98), whereas 5-20% of circulating lymphocytes appear to be NK cells in humans (99). However, other publications still observed an important NK-like activity in chickens (73, 100). One of the most plausible explanations resides in the difficulty to properly identify them in chickens. Indeed, in contrast to B and T cells, NK cells do not appear to have a unique specific marker. It is possible that the frequency of NK cells is far higher in peripheral tissues, and that the panel of markers used in the above-mentioned study (TCR^-^ IgL^-^ CD3^-^ CD8^+^ lymphocytes) may be inappropriate or insufficient (86). There is therefore a need to identify additional markers for NK cells, like the analog of CD56 that is used to identify NK cells in humans and chickens (101, 102). Another explanation could be an actual lack of NK cells, compensated with other functionally similar T cells. For example, the high proportion of γδ T cells in chickens, as described further below, might potentially be involved in maintaining an NK-like activity, since they share common features with NK cells (103, 104). Further studies are then needed to properly understand how much NK cells contribute to the chicken immune system.

#### 2.2.5 Other Innate and Related Immune Cells

While all the above-mentioned classical immune cells play essential and distinctive roles in the general immune response, other cells are also involved. Among them, we find the mast cells, described as tissue-resident hematopoietic cells with cytoplasmic granules. In humans, these cells are capable of histamine release and are involved in a wide range of physiological processes, including homeostasis, tissue repair, and angiogenesis. Additionally, they are associated with innate and adaptive immune processes, including immune tolerance and inflammation (105–107). Even though they seem to be quite similar to basophils, in both structure and function, they differ in their development, their reaction to stimuli, and their ability to release immunomodulating cytokines (108). In chickens, mast cells have been greatly studied. Many aspects, including the general ontogeny, the morphology, and the biological functions, appear to be similar to their mammalian analogs (109). Several studies have also demonstrated that avian mast cells can mediate the inflammatory response, infiltrate contaminated tissues and release antimicrobial substances after pathogen infection, similar to human mast cells (110, 111).

In the circulating blood, two other essential types of cells can be found: the thrombocytes, and the erythrocytes. They are respectively involved in coagulation (112) and oxygen delivery (113), though they also appear in humans to be both potentially involved in the mediation of immune responses (114–116). While human thrombocytes and erythrocytes are both enucleated, they appear different in chickens as they kept their nucleus. Many aspects, such as morphology, biology, and ontogeny, are different in both species. For instance, avian thrombocytes arise from a stem cell whereas mammalian thrombocytes arise from megakaryocytes (112, 117). In chickens, thrombocytes have a more defined role in the immune response: even though they share similar biological functions to their mammalian equivalents (*e.g*., initiation of coagulation), various papers report how their role in the mediation of the inflammatory response is important. Indeed, thrombocytes are not only able to recognize pathogen-associated molecular patterns through Toll-like receptors (TLRs), but can also perform phagocytosis, produce nitric oxide, and release pro-inflammatory cytokines (118–120). Likewise, in contrast to their mammalian counterparts, avian erythrocytes have conserved their nucleus and mitochondria, allowing the cell to produce reactive oxygen species that might be used as an antimicrobial defense (121). Nucleated erythrocytes are believed to have a direct role in immune responses, as they also express TLRs and are capable of regulating the expression of different immune-related genes (*e.g.*, TLRs, CCL4, and α-interferon) (122). Thus, thrombocytes and erythrocytes are believed to play a more important role in the chicken immune response than their mammalian counterparts.

#### 2.2.6 T Cell Lineage, From Helpers to Effector Lymphocytes

T cells are a key component of the cell-mediated adaptive immune response and can be distinguished from other lymphocytes by the presence of T cell receptors (TCR) on their surface. They are differentiated from hematopoietic progenitors and matured in the thymus, before being released into the bloodstream. These immune cells are then able to recognize antigens presented by major histocompatibility complex (MHC) molecules through their TCR. T cell development and conservation between avian and mammalian models have been investigated for more than two decades and are now well-described: central features, such as the general TCR structures, the T cell subpopulations, and their respective biological functions, seem to be similar between chickens and humans (123, 124).

As in humans, chicken T cells use the TCR, a heterodimeric surface receptor, for antigen recognition. Each TCR chain is composed of two immunoglobulin super-family domains: a variable region with an extensive sequence diversity, and a constant domain, anchored into the plasma membrane. Both birds and mammals have similar TCR chains which can form either TCR-αβ or TCR-γδ heterodimers, defining the two major T cell lineages. TCR-αβ^+^CD4^+^ T cells are often described as T helper cells, TCR-αβ^+^CD8^+^ T cells as cytotoxic T cells, and TCR-γδ^+^ T cells as a cytotoxic lymphocyte subset that can bind to many different ligands, independently of MHC recognition (124–127). γδ T cells are mainly enriched in peripheral tissues, where they make key contributions to immune responses with essential functions such as immune surveillance and protection that cannot be compensated by αβ T cells (128, 129). Overall, the TCR complexes are quite similar between humans and chickens, and their assembly, their surface expression, and their signal transduction are all controlled and regulated by CD3, a transmembrane receptor (124, 130).

Despite the similarity in T cell structures and functions, there are interesting differences between the two species as well. For instance, in mammals, the CD3 complex consists of three chains: CD3γ, CD3δ, and CD3ϵ. These chains can then form two different heterodimers, CD3ϵγ and CD3ϵδ, before assembly with the TCR. In chickens, however, only two CD3 genes exist: a CD3ϵ homolog, and a single CD3γ/δ gene, which has an equal homology to both mammalian CD3γ and CD3δ. Both subunits form two identical dimers (CD3ϵγ/δ) before complex assembly (124, 131). This difference might be explained by CD3γ/δ being an evolutionary precursor before CD3γ and CD3δ genes were split in mammals. Even though CD3 complexes share similar functions in both species, chicken TCR/CD3 components are not able to replace the human molecules in T cells, whereas this is possible for sheep and mouse TCR chains (132). Another notable difference between the two species resides in the TCR expression. Indeed, while γδ T cells have a relatively low expression in humans (around 2 to 10%), frequencies of γδ T cells in chickens can reach up to 50% of the circulating T cell population. This can be explained by the fact that chicken γδ T cells develop exclusively in the thymus, whereas development in mammals can also occur in periphery organs where this T cell subpopulation is enriched (124, 126, 128, 133).

#### 2.2.7 B Cell Lineage and the Antibody Repertoire Generation

While most T cells’ functions are related to cell-mediated immunity, B cells are instead mainly known to be involved in the humoral component of the adaptive immune system through the production of antigen-specific antibodies, a secreted form of the B cell receptors (BCR), following exposure to a pathogen. B cells, and the generation of the BCR repertoire, have been conserved throughout evolution and are relatively similar between chickens and humans (134). Even though the cells themselves share a great resemblance to both species, the main difference resides in their maturation. As mentioned earlier, while B cells mature in the bone marrow in humans, their development in chickens occurs in an avian-specific organ: the bursa of Fabricius. This organ’s function has been discovered as early as 1956 through a series of bursectomy experiments on chickens, where Glick et al. demonstrated that the antigen-specific antibody response was bursa-dependent (135). The general nature and development of B cells, analogous between humans and chickens despite the different organs, have then been thoroughly described in the literature (136, 137).

Succinctly, key differences between the two species are linked to the generation of the antibody repertoire. Indeed, in mammals, this process happens mostly through gene rearrangement, which is ongoing throughout life. Each chain of immunoglobulins, heavy and light, is composed of a V (variable) region, a D (diversity) region for heavy chains, and J (joining) segments, joined together with a C (constant) region to produce functional immunoglobulins with VJ light chains and VDJ heavy chains. This production of variable regions leads to a vast antibody repertoire of more than 10^11^ different immunoglobulins that can be generated (31, 137, 138). This process is different in chickens due to their very limited number of variable genes. Because they only have a single copy of functional V and J segments for both chains, the chicken immune system relies on another mechanism known as somatic gene conversion, a process taking place during bursal development (134, 136). Thus, the clusters of pseudogenes, upstream of the immunoglobulin loci, are critical for antibody diversity in chickens (139, 140). After a low-efficiency V(D)J rearrangement in the bone marrow, immature B cells migrate to the bursa of Fabricius where gene conversation, a process in which V sequences are replaced with the pseudogene sequences, takes place. This contributes to the chickens’ robust immune response, with a mammalian-like BCR repertoire (31). However, while the generation of the mammalian antibody repertoire continues throughout life, gene conversion in chickens is only active until the bursa involutes, around 6 months after hatching. It is believed that afterwards, the adult chicken might use, as a source of B cells, post-bursal stem cells mainly from the spleen (31, 134, 136).

Finally, while chicken and mammalian immunoglobulins are also similar in their general structure and functionality, there are notable differences between the two species as well. Indeed, there is some confusion in the literature: although chickens are not able to produce IgG *per se*, they do secrete another immunoglobulin, IgY. Mammalian IgG and avian IgY functions are equivalent, but the main difference resides in their structure: the chicken IgY heavy chain contains one V domain and four C domains, whereas mammalian IgG contains only three C domains (136, 141). Furthermore, even though chickens have homologs for mammalian IgM and IgA, they are not able to generate IgD and IgE at all. It is nonetheless believed that mammalian IgE functions may be replaced by avian IgY (142, 143).

### 2.3 Key Receptors and Molecules on Immune Cells

To properly function, immune cells need essential proteins at their surface, which are useful for recognizing and presenting antigens, co-stimulating effector proteins, and regulating inflammation. While some of these proteins have already been described in this review, others also play essential functions in the immune system overall and ought to be detailed further. For instance, to recognize pathogen-associated molecular patterns, immune cells, namely antigen-presenting cells and granulocytes, express pattern-recognition receptors at their surface. To this day, some of the best-characterized ones are TLRs, which have been thoroughly described both in humans and chickens (73, 144, 145). Kaiser et al. have detailed the similarities and differences of the TLRs between both species (47, 77). They have mainly shown that chickens have a different repertoire of TLRs: some chicken TLRs have human orthologs (*e.g.*, TLR4), some possess slight variations (*e.g.*, human TLR2 ortholog is duplicated into TLR2A and TLR2B in chickens), some human TLRs are absent (*e.g.*, TLR9), and some are specific to the chicken (TLR15 and TLR21). However, despite the different repertoire, chicken cells can potentially recognize a range of pathogens similar to mammals’ (146).

Another essential cell-surface protein present in most cells, and specifically in the antigen-presenting cells, are the major histocompatibility complexes (MHC). MHC class I molecules are expressed by all nucleated cells and can present exogenous antigens to cytotoxic T lymphocytes. MHC class II molecules, however, are expressed by B cells, dendritic cells, and macrophages, and they process antigen material to T helper cells. While phagocytosis and endocytosis are highly similar between chickens and humans, the MHC molecules are however somewhat different. Indeed, the main difference is that compared to its mammalian counterpart, the chicken MHC is very compact and less polymorphic: the human MHC contains over 200 genes in a region of around 4000 kb, while the chicken MHC contains only 46 genes in a region of approximately 209 kb (147–149). Nevertheless, the chicken MHC contains the essential counterparts of genes present in the mammalian MHC, with two class I genes and two class IIβ genes, allowing the animal to potentially mount a robust, yet inferior, immune response to a pathogen (27, 150).

Finally, some of the most studied cell-surface proteins for these last couple of years, especially for cancer treatment, are immune checkpoints. Indeed, most of the therapeutic strategies currently developed involve immune checkpoint inhibitors, a revolutionary approach allowing reactivation of the immune system and generation of an efficient antitumor immune response (151). In humans, many of these proteins have been described, and some of them are already targeted by marketed treatments: among them, we find PD-1, PD-L1, CTLA-4, LAG-3, A2aR, BTLA, IDO, and KIR (152). For all of these proteins, homologs have been found and described in chickens, highlighting a great similarity between both species (88, 153–157).

### 2.4 Cytokines and Chemokines

Cytokines and chemokines are small, secreted proteins involved in the growth, differentiation, and activation of the immune cells. Cytokines’ main functions are to elicit and regulate immune responses, whereas chemokines are mostly involved in controlling the traffic of immune cells. They are both crucial to immune defense and homeostasis, and they both help modulate inflammatory responses and organize the cellular arrangement of immune organs (158, 159). Among the vast repertoire of human cytokines and chemokines, a high proportion has been identified to be present in chickens as well, though some exceptions remain. For instance, in multigene families, chickens seem to have fewer members than their mammalian counterparts. The Tumor Necrosis Factors superfamily and their receptors, for instance, lack several mammal orthologs in chickens, though functional orthologs for TNF-α and other cytokines can be found (57, 160). Likewise, while the interleukin-1 family is composed of 11 cytokines in humans, only four (IL-1β, IL-18, IL-1RN, and IL-36RN) have been found in chickens to date, with similar biological functions (78, 161). Chicken cytokines have in general only about 25-35% of amino acid identity with their mammalian orthologs, although they share similar biological activities (27, 78). Because of the vastness of the human cytokine repertoire, this review will not detail every point of comparison with the chicken’s. Avian cytokines have already been thoroughly described in the literature, and have been compared in great detail with their mammalian counterparts (78, 161, 162).

### 2.5 Inflammation

Inflammation is a complex adaptive response that can be triggered by various harmful stimuli, such as an infection, a tissue injury, or a tissue malfunction. This process involves most of the immune system, and its main functions are to eliminate the cause of inflammation (*e.g.*, pathogens, damaged cells, irritants), clear out necrotic cells, and repair damaged tissues (163). Inflammation can generally be split into two phases. The first one is an acute phase, where immune cells, mainly granulocytes, migrate to the site of injury, remove the inflammatory stimulus, and eventually initiate healing. Acute inflammation is often enough to eliminate the cause of inflammation. However, if it persists, it can eventually reach a chronic phase, where immune cells are continually being recruited. While acute inflammation mainly promotes immune defense, chronic inflammation essentially supports tissue repair characterized by the accumulation of tissue macrophages and fibroblasts (164). However, chronic inflammation can sometimes lead to various complications, like tissue damage and fibrosis, which can cause and amplify a wide range of pathologies, including cardiovascular diseases, diabetes, and cancer (1, 165, 166).

All the immune cells mentioned until now play essential roles in the coordination of inflammation. They all make use of a vast panel of mediators to precisely orchestrate this process through various signaling pathways (167, 168). Among them, we find many cytokines and chemokines that act as soluble mediators to regulate inflammation. Some appear to be essential for proper mediation. Indeed, in humans, IL-1β, IL-6, IL-8, IL-12, IL-17, IFN-γ, TNF-α, and GM-CSF have all been reported to be key promotors for the activation of inflammation while other cytokines, such as IL-4, IL-10, and TGF-β, promote inhibition (159, 167, 168). In chickens, homologs for all these cytokines and chemokines have been found and documented, with biological functions similar to their mammalian equivalents (57, 66, 169–176). The same is true with downstream inflammatory mediators, such as iNOS, COX-2, and prostaglandin (168, 177). Another key factor for the mediation of inflammation is the complement system that has also been thoroughly described in chickens, with high similarity to its human equivalent (73, 178).

Classical animal models of inflammation use the administration of lipopolysaccharide (LPS). This large molecule is a specific component of the outer membrane of Gram-negative bacteria and can induce an inflammatory response *via* TLR4 (179, 180). Many studies have been done in chickens, where administration of LPS has been shown to cause a significant influx of heterophils, and to increase the release of pro-inflammatory mediators such as IL-1β, IL-6, IL-8, TNF-α, iNOS, and COX-2 (145, 181–183). Other methods, such as ammonia exposure, can also induce inflammation in the chicken model due to its irritant properties (184).

## 3 The Chicken Embryo as a New Paradigm for Immune-Based Studies

**Figure 2:**
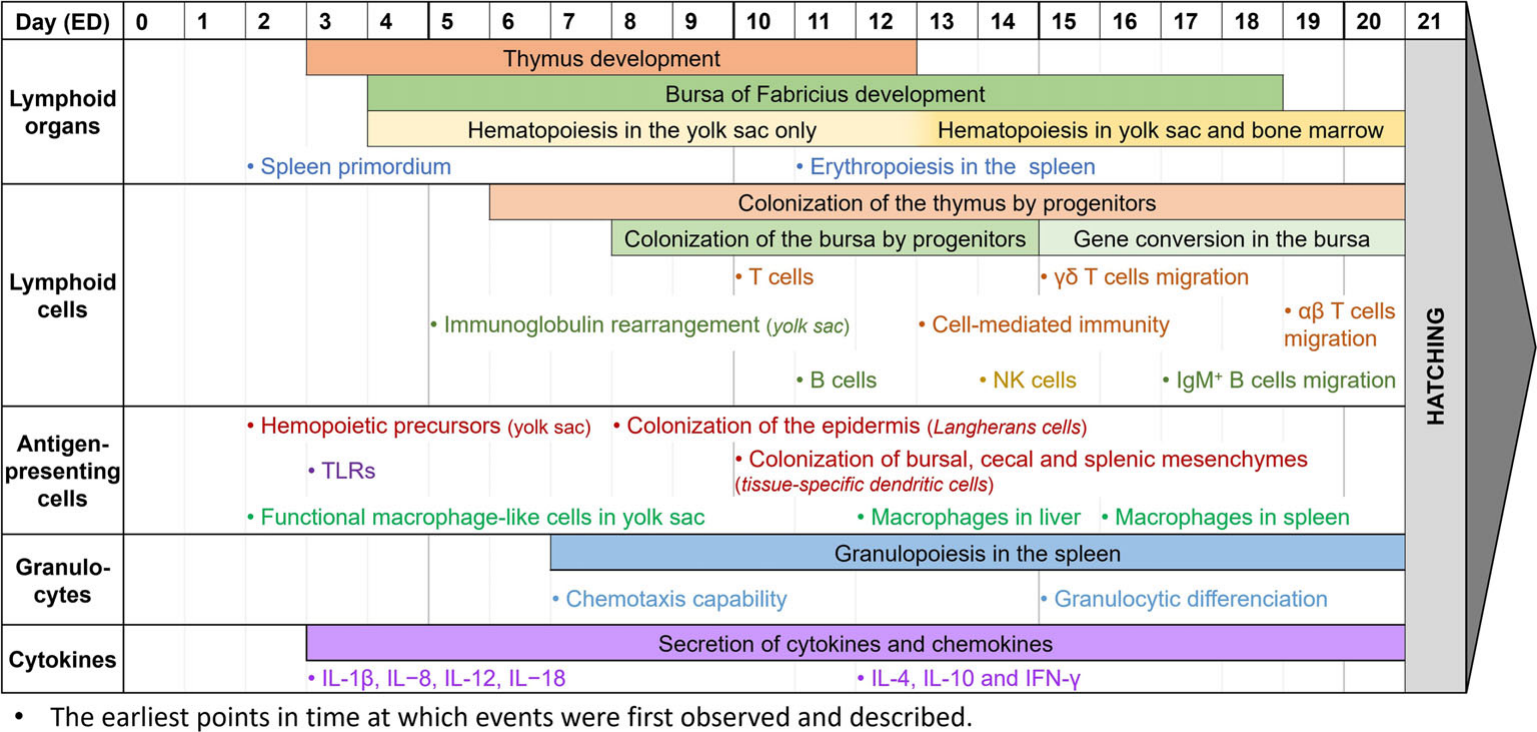
Observed immune system features during chicken embryogenesis. Caveat: This figure summarizes observations reported in the scientific literature. They correspond to specific studies each performed at specific time points and do not represent the extent of immune system features over the embryogenesis. Thus, it cannot be interpreted as an accurate depiction of the immune system development in ovo, since most papers do not cover the entire development period.

The chicken embryo model shows many advantages over other classical models such as rodents. Besides being a partial solution for the ethical issues raised earlier, this model is also simpler to maintain, gives faster results with a lower cost, and can be used to screen a large range of molecules and biotherapies. Furthermore, the model is characterized by high reproducibility and reliability, and its biology and physiology are well-known. However, the chicken embryo model still has some limitations. For instance, the reagents for immune-based studies are limited compared to the mouse model, the protocols are less standardized, and it is not possible to orally administrate drugs (9, 11, 16). Nevertheless, before embryonation day (ED) 9, the chicken embryo develops only partial immunity, allowing the model to be grafted with human cells with a low risk of transplant rejection, while gradually building its immunocompetence (9, 11, 133). As some components of the innate immune system still develop after hatching, the immune responses of late-stage chicken embryos might be sufficient, yet incomplete (185). To this date, it is still difficult to properly describe the ontogeny of the immune system in chickens. Many papers try to document the development of avian immunology (133, 186–189), but some information can be contradictory and the general timeline lacks precision. Thus, to determine whether the chicken embryo could be an interesting model for immune-based studies, it is essential to perform a general overview of immune system development *in ovo* (**Figure 2**). Most of the information described below comes from general observations from the scientific literature. As such, further investigation is needed to precisely determine the various time points of *in ovo* immune system development. We kindly remind the reader that some of the time points described below might not accurately represent the complete chronology of immune development and is instead an observation of existing immune components at different times. Some of the immune components described may have already appeared earlier in the chicken embryogenesis.

During the embryonic development of chickens, the first lymphoid organ to develop is the thymus. The thymic rudiment first appears at ED3, and the organ is fully developed by ED12 (186, 189). The epithelial anlage of the bursa of Fabricius usually appears a bit later, at ED4-5, then undergoes several structural transformations until ED14, when epithelial cells transform into lymphoid cells. By ED18, about three days before hatching, the bursa has a well-organized lymphoid structure (27, 139, 186, 189). Since the bone marrow is still being developed in the early stages of embryogenesis, hematopoiesis occurs only in the yolk sac between ED4 and ED12, then in both organs after ED13 (190, 191). Finally, during embryo development, the spleen has a high contribution to lymphopoiesis. Its primordium first appears at ED2, hosts granulopoiesis from ED7, erythropoiesis from ED11, and some B cell progenitors undergo BCR rearrangement there before colonizing the bursa. It only becomes a secondary lymphoid organ once the egg hatches (27, 29).

While the lymphoid organs are being formed, lymphoid cells undergo development as well. Common lymphoid progenitors, deriving from the yolk sac and spleen, colonize the epithelial thymus in three waves: at ED6, ED12, and ED18, until just after hatching (186, 189). T cells can be detected at ED10-11, and cell-mediated immunity has been reported as soon as ED13-14. T cells are then believed to be fully immunocompetent by ED18 (14, 192, 193). Among them, γδ T cells are the first to appear in the thymus, three days before αβ T cells (133, 186, 189). After maturation and TCR rearrangement, γδ T cells migrate to the spleen and intestinal epithelium by ED15, whereas αβ T cells only start doing so at ED19 (27, 133, 194, 195). Meanwhile, the bursa of Fabricius rudiment is colonized by lymphoid progenitors between ED8 and ED14 (136, 140, 186). B cells can first be detected there at ED11-12, and like T cells, are believed to be fully immunocompetent by ED18 (14, 193, 196). Immunoglobulin gene rearrangement begins before colonization of the bursa, and can be detected as early as ED5-6 in the yolk sac, and only at about ED10 in the bursa (197). However, until ED15, B cell progenitors isolated from the bursa mostly had partial or non-productive gene rearrangements (136). Gene conversion initiates later in developing B cells at ED15-17, until bursal involution, about 6 months after hatching (186). This allows B cells to produce a wider panel of antigen-specific immunoglobulins, as explained earlier. Immature IgM^+^ cells can be detected at ED12 when lymphoid progenitors colonize the bursa, and B cells are then able to produce IgM at ED14 and IgY at ED21, around hatching time (186, 189). B cells gradually mature between ED16 and ED19, with great differences in the genes expressed, until they leave the bursa with rearranged BCRs (27, 198). IgM^+^ B cells are already observed outside the bursa at ED17, and IgY^+^ B cells are only detected in other organs post-hatch (186). NK cells, the third lymphoid cell population, are generated from the bone marrow and can be detected in the embryonic spleen at ED14. At this time, they are already functionally similar to those from 4-week-old chickens (86, 199). However, NK cell functionality might appear earlier. Indeed, since these studies only begin at ED14, it is unsure from which time point embryonic NK cells can be detected, and when they gain their functionality. After T cell migration from the thymus, NK cells are found in abundance both in the intraepithelial lymphocyte compartment and the embryonic spleen by ED19 (87, 133).

Antigen-presenting cells, and more generally CD45^+^ cells, are derived from progenitors from the bone marrow in mature chickens. However, at the early stages of embryonic development, they are derived from the yolk sac instead, and precursors can be found as early as ED2 (200). While avian dendritic cells and their ontogeny are not well-known yet, Dóra et al. have found that CD45^+^ MHC II^+^ cells colonize the epidermis from ED8, representing potential precursors of Langerhans cells. They have also demonstrated that these cells colonize the bursa of Fabricius, the cecal mesenchyme, and the splenic mesenchyme at about ED10, and might then differentiate into other tissue-specific dendritic cells (200). Other studies have shown that thymic dendritic cells precursors are identified in the thymus by ED11 (45). It is however unsure when these precursors mature into dendritic cells and become functionally competent. Macrophage development, however, is better described in chicken embryogenesis. In the early stages of development, at about ED2-4, cells with a macrophage-like morphology and a phagocytic activity can already be found in the yolk sac. They appear to be homologs of mammalian fetal macrophages, and they may play a crucial role in tissue growth and remodeling by removing apoptotic cells (46, 133). Later, macrophages can also be observed in the liver at ED12, and in the spleen at ED16 (46, 187, 193). They are functionally competent, as they can recognize and phagocytose microbial antigens, and they are capable of chemotaxis. However, they are not recruited to incisional wounds during embryonic development (17, 133, 201). Most TLRs, essential for antigen-specific recognition, are expressed as early as ED3 in chicken embryos. Their expression increases over time, especially in the liver where TLR4 is highly expressed at ED12, which might be correlated to the progressive acquisition of the chicken’s immunocompetence (133, 202).

To date, knowledge of avian granulocyte ontogeny is lacking, and the structural and functional developments *in ovo* are not properly defined. Granulopoiesis appears to begin early in embryonic development, with an expansion of the granulocytic lineages from ED7 to ED20 in the yolk sac and the splenic primordium, and granulocytic differentiation in the liver from ED15 (29, 190). Reports on heterophil functionality are usually done once the egg has already hatched, and they describe how their activity, including phagocytic ability, microbial killing, degranulation, and oxidative burst, appears decreased compared to mature birds until 21 days of age (75, 133). It is however unsure when heterophils begin to be functional. Early studies have demonstrated that chicken embryos, stimulated at ED18, have heterophils with enhanced immune functions, suggesting that they are already functional at that time (203, 204). Furthermore, high recruitment of heterophils to an inflammatory site can be detected as early as ED7 (205). Xenografts of foreign tumor cells in the model have also shown that the chicken embryo is not able to mount a proper immune response until ED12, though there is a visible infiltration of avian heterophils in the tumor microenvironment (11). Overall, this suggests that chicken heterophils are capable of chemotaxis early in development, and while it is unsure when they become immunocompetent, they appear to be functional between ED12 and ED18. Mast cells are first detected at around ED3 in the chicken extraembryonic vascular membranes, and at around ED14 in the uvea and the lung while they gradually become functional (109). After hatching, the number of mast cells greatly increases, especially in the bursa of Fabricius in 7-day-old chickens and the thymus and spleen in 21-day-old chickens (206).

Even though most cytokines are expressed at high levels two weeks after hatch (207), they can already be detected in the chicken embryo, as reviewed by Alkie et al. (133). IL-1β, IL−8, IL-12, and IL−18 can be detected as early as ED3 (208) while IL-4, IL-10, and IFN-γ are identified in the spleen at ED12 (209). It is not clear when IL-6 begins to be expressed but late-stage studies at ED18 were able to quantify its expression (185). The early secretion of cytokines suggests that the model can be used to study inflammation. Several papers have demonstrated that this model, and the use of its CAM, can be pertinent for inflammation studies (17, 210–212). Indeed, deposits of LPS onto the CAM as early as ED7 have been shown to induce a significant inflammatory response after 24 hours (205). Other studies at ED9-11 have described that the presence of TLR ligands, including LPS and Pam3CSK4, upregulates the expression of pro-inflammatory cytokines (213). Finally, intestinal epithelial cells show enhanced expression of IL-6 and IL-18 at ED17 after LPS administration (214). All these studies show how, even with a partial immune system, the chicken embryo is already capable of mounting a robust immune response, making this model pertinent for immune-based studies. Thus, correct timing is extremely important for addressing immune-specific questions, and experiments requiring a more complex immune system should be performed at the appropriate stage (**Figure 2**).

Besides inflammatory studies, CAM assays are used for a large range of biological studies. Because of their dominant advantages compared to other models, they are now widely used for studying the angiogenesis process *in vivo* (215, 216). Many papers have described its relevance for basic angiogenesis studies. Indeed, its rich vascular network, high oxygenation, and rapid development are ideal for investigating cardiovascular diseases (217), retinopathy (17), and anti-angiogenetic treatments (218). Moreover, the CAM is used to investigate tumor-related angiogenesis. Since the chicken embryo’s immune system develops gradually over time, it is possible to perform tumor tissue xenografting while the immune system is still immature. Previous studies have confirmed that grafting before the first observed T cells in the thymus at ED10-11 offers a lower rejection rate (14, 192, 215, 219). It is however important to keep in mind that because of the progressive development of its immunocompetence, non-specific inflammatory reactions can occur if experimentation on the CAM is performed at a later embryonic stage (9, 11, 215). To conclude, the CAM is now described as an excellent platform for tumor xenografting, and for understanding the tumor-related angiogenesis process (18, 220–222). More importantly, it can be used to investigate the efficacy and mechanisms of action of proangiogenic and antiangiogenic agents (13, 223).

## 4 Discussion

Even after more than 310 million years since the last common ancestor, chickens and humans, living in the same environmental niche and challenged with the same ranges of pathogens (27, 224), appear to be capable of building comparable immune responses. Indeed, despite the different lymphoid organs and even though some key differences should be highlighted, chickens appear to have a simpler yet functionally similar immune system. The chicken model has already enabled valuable contributions to our understanding of immunology and is now considered to be excellent for immune-based studies (31). More recently, the use of the chicken embryo model has re-emerged and has shown many strong advantages over other classical models, including cost-effectiveness, time efficiency, and simplicity. It can be used in a multitude of applications, such as the evaluation of angiogenesis, tumor growth, metastasis, and therapy responses. However, this model also has limitations and cannot be suggested as a complete replacement of classical preclinical models. It can instead be used as an intermediate step between cell culture and a more complex mammalian model, following the general principles of the 3Rs (9, 205). In this review, we described how the chicken embryo could build a robust immune response even with a partial immune system, making this model pertinent for immune-based studies, mainly after 10 days of embryonic development (225). However, because the avian immune system appears to be partially different from humans, all discrepancies in immune responses should be considered when using this model. Despite these caveats, the model appears to be an excellent way to study immunology and inflammation and can be an optimal solution for validating various ranges of molecules.

## Author Contributions

PG, YW, JV, and ZM contributed to the conception and design of the review. PG wrote the first draft of the manuscript. All authors contributed to manuscript revision, read, and approved the submitted version.

## Funding

PG was supported by a CIFRE PhD fellowship funded in part by ANRT (National Association for Research and Technology) on behalf of the French Ministry of Higher Education (grant n°2020/1519). This work was partially funded by the French Région Auvergne Rhône Alpes’s R&D Booster program, through the “InovoFlammTest” project.

## Conflict of Interest

PG, YW, and JV are employees of Inovotion.

The remaining author declares that the research was conducted in the absence of any commercial or financial relationships that could be construed as a potential conflict of interest.

## Publisher’s Note

All claims expressed in this article are solely those of the authors and do not necessarily represent those of their affiliated organizations, or those of the publisher, the editors and the reviewers. Any product that may be evaluated in this article, or claim that may be made by its manufacturer, is not guaranteed or endorsed by the publisher.

## References

[1] Furman D Campisi J Verdin E Carrera-Bastos P Targ S Franceschi C. Chronic Inflammation in the Etiology of Disease Across the Life Span. Nat Med (2019) 25:1822–32. doi: 10.1038/s41591-019-0675-0 PMC7147972 31806905

[2] Dolan RD McMillan DC. The Prevalence of Cancer Associated Systemic Inflammation: Implications of Prognostic Studies Using the Glasgow Prognostic Score. Crit Rev Oncol Hematol (2020) 150:102962. doi: 10.1016/j.critrevonc.2020.102962 32344318

[3] Cox AJ West NP Cripps AW. Obesity, Inflammation, and the Gut Microbiota. Lancet Diabetes Endocrinol (2015) 3:207–15. doi: 10.1016/S2213-8587(14)70134-2 25066177

[4] El-Gabalawy H Guenther LC Bernstein CN. Epidemiology of Immune-Mediated Inflammatory Diseases: Incidence, Prevalence, Natural History, and Comorbidities. J Rheumatol Suppl (2010) 85:2–10. doi: 10.3899/jrheum.091461 20436161

[5] Carbone L. Estimating Mouse and Rat Use in American Laboratories by Extrapolation From Animal Welfare Act-Regulated Species. Sci Rep (2021) 11:493. doi: 10.1038/s41598-020-79961-0 33436799 PMC7803966

[6] O’Connor J Lawson M Andre C Moreau M Lestage J Castanon N. Lipopolysaccharide-Induced Depressive-Like Behavior is Mediated by Indoleamine 2,3-Dioxygenase Activation in Mice. Mol Psychiatry (2009) 14:511–22. doi: 10.1038/sj.mp.4002148 PMC2683474 18195714

[7] Noh H Jeon J Seo H. Systemic Injection of LPS Induces Region-Specific Neuroinflammation and Mitochondrial Dysfunction in Normal Mouse Brain. Neurochem Int (2014) 69:35–40. doi: 10.1016/j.neuint.2014.02.008 24607701

[8] Mestas J Hughes CCW. Of Mice and Not Men: Differences Between Mouse and Human Immunology. J Immunol Baltim Md 1950 (2004) 172:2731–8. doi: 10.4049/jimmunol.172.5.2731 14978070

[9] Schneider-Stock R Ribatti D. The CAM Assay as an Alternative In Vivo Model for Drug Testing. In: Schäfer-Korting M Stuchi Maria-Engler S Landsiedel R, editors. Organotypic Models in Drug Development Handbook of Experimental Pharmacology. Cham: Springer International Publishing (2020), 303–23. doi: 10.1007/164_2020_375 32776283

[10] Dünker,J. Implementation of the Chick Chorioallantoic Membrane (CAM) Model in Radiation Biology and Experimental Radiation Oncology Research. Cancers (2019) 11:1499. doi: 10.3390/cancers11101499 PMC6826367 31591362

[11] Ribatti D. The Chick Embryo Chorioallantoic Membrane (CAM). A Multifaceted Experimental Model. Mech Dev (2016) 141:70–7. doi: 10.1016/j.mod.2016.05.003 27178379

[12] Achkar IW Kader S Dib SS Junejo K Al-Bader SB Hayat S. Metabolic Signatures of Tumor Responses to Doxorubicin Elucidated by Metabolic Profiling in Ovo. Metabolites (2020) 10:268. doi: 10.3390/metabo10070268 PMC7408021 32605263

[13] El Hasasna H Saleh A Samri HA Athamneh K Attoub S Arafat K. Rhus Coriaria Suppresses Angiogenesis, Metastasis and Tumor Growth of Breast Cancer Through Inhibition of STAT3, Nfκb and Nitric Oxide Pathways. Sci Rep (2016) 6:21144. doi: 10.1038/srep21144 26888313 PMC4758048

[14] Kundeková B Máčajová M Meta M Čavarga I Bilčík B. Chorioallantoic Membrane Models of Various Avian Species: Differences and Applications. Biology (2021) 10:301. doi: 10.3390/biology10040301 33917385 PMC8067367

[15] Marcion G Hermetet F Neiers F Uyanik B Dondaine L Dias AMM. Nanofitins Targeting Heat Shock Protein 110: An Innovative Immunotherapeutic Modality in Cancer. Int J Cancer (2021) 148(12):ijc.33485. doi: 10.1002/ijc.33485 33506516

[16] Nowak-Sliwinska P Segura T Iruela-Arispe ML. The Chicken Chorioallantoic Membrane Model in Biology, Medicine and Bioengineering. Angiogenesis (2014) 17:779–804. doi: 10.1007/s10456-014-9440-7 25138280 PMC4583126

[17] Rezzola S Loda A Corsini M Semeraro F Annese T Presta M. Angiogenesis-Inflammation Cross Talk in Diabetic Retinopathy: Novel Insights From the Chick Embryo Chorioallantoic Membrane/Human Vitreous Platform. Front Immunol (2020) 11:581288. doi: 10.3389/fimmu.2020.581288 33117388 PMC7552803

[18] Ribatti D Tamma R. The Chick Embryo Chorioallantoic Membrane as an In Vivo Experimental Model to Study Multiple Myeloma. In: The Enzymes. Bari, Italy: Elsevier (2019), 23–35. doi: 10.1016/bs.enz.2019.08.006 31727275

[19] Rous P Murphy JB. Tumor Implantations in the Developing Embryo. J Am Med Assoc (1911) LVI:741–2. doi: 10.1001/jama.1911.02560100033015

[20] Murphy JB. Transplantability of Tissues to the Embryo of Foreign Species : Its Bearing on Questions of Tissue Specificity and Tumor Immunity. J Exp Med (1913) 17:482–93. doi: 10.1084/jem.17.4.482 PMC2125042 19867659

[21] Boehm T Hess I Swann JB. Evolution of Lymphoid Tissues. Trends Immunol (2012) 33:315–21. doi: 10.1016/j.it.2012.02.005 22483556

[22] Boehm T Swann JB. Origin and Evolution of Adaptive Immunity. Annu Rev Anim Biosci (2014) 2:259–83. doi: 10.1146/annurev-animal-022513-114201 25384143

[23] Franchini A Ottaviani E. Thymus: Conservation in Evolution. Gen Comp Endocrinol (2017) 246:46–50. doi: 10.1016/j.ygcen.2017.03.011 28322763

[24] Zlotoff DA Bhandoola A. Hematopoietic Progenitor Migration to the Adult Thymus. Ann N Y Acad Sci (2011) 1217:122–38. doi: 10.1111/j.1749-6632.2010.05881.x PMC3076003 21251013

[25] Yu VWC Scadden DT. Hematopoietic Stem Cell and Its Bone Marrow Niche. Curr Top Dev Biol (2016) 118:21–44. doi: 10.1016/bs.ctdb.2016.01.009 27137653 PMC6854531

[26] van Ewijk W Wang B Hollander G Kawamoto H Spanopoulou E Itoi M. Thymic Microenvironments, 3-D Versus 2-D? Semin Immunol (1999) 11:57–64. doi: 10.1006/smim.1998.0158 9950752

[27] Kaiser P Balic A. The Avian Immune System. In: Sturkie’s Avian Physiology. Edinburgh, Scotland, UK: Elsevier (2015). pp. 403–18. doi: 10.1016/B978-0-12-407160-5.00017-8

[28] Thapa P Farber DL. The Role of the Thymus in the Immune Response. Thorac Surg Clin (2019) 29:123–31. doi: 10.1016/j.thorsurg.2018.12.001 PMC6446584 30927993

[29] Oláh I Nagy N Vervelde L. Structure of the Avian Lymphoid System. In: Avian Immunology. Budapest, Hungary: Elsevier (2014). pp. 11–44. doi: 10.1016/B978-0-12-396965-1.00002-9

[30] Ifrah ME Perelman B Finger A Uni Z. The Role of the Bursa of Fabricius in the Immune Response to Vaccinal Antigens and the Development of Immune Tolerance in Chicks (Gallus Domesticus) Vaccinated at a Very Young Age. Poult Sci (2017) 96:51–7. doi: 10.3382/ps/pew232 27418658

[31] Davison F. The Importance of the Avian Immune System and its Unique Features. In: Avian Immunology. UK: Elsevier (2014). pp. 1–9. doi: 10.1016/B978-0-12-396965-1.00001-7

[32] Glick B. The Bursa of Fabricius: The Evolution of a Discovery. Poult Sci (1994) 73:979–83. doi: 10.3382/ps.0730979 7937486

[33] Madej JP Chrząstek K Piasecki T Wieliczko A. New Insight Into the Structure, Development, Functions and Popular Disorders of Bursa Fabricii. Anat Histol Embryol (2013) 42:321–31. doi: 10.1111/ahe.12026 23438192

[34] Siatskas C Boyd R. Regulation of Chicken Haemopoiesis by Cytokines. Dev Comp Immunol (2000) 24:37–59. doi: 10.1016/S0145-305X(99)00051-8 10689097

[35] Brand A Galton J Gilmour DG. Committed Precursors of B and T Lymphocytes in Chick Embryo Bursa of Fabricius, Thymus, and Bone Marrow. Eur J Immunol (1983) 13:449–55. doi: 10.1002/eji.1830130604 6190659

[36] Buettner M Bode U. Stromal Cells Directly Mediate the Re-Establishment of the Lymph Node Compartments After Transplantation by CXCR5 or CCL19/21 Signalling. Immunology (2011) 133:257–69. doi: 10.1111/j.1365-2567.2011.03436.x PMC3088987 21426341

[37] Stebegg M Kumar SD Silva-Cayetano A Fonseca VR Linterman MA Graca L. Regulation of the Germinal Center Response. Front Immunol (2018) 9:2469. doi: 10.3389/fimmu.2018.02469 30410492 PMC6209676

[38] Oláh I Kupper A Kittner Z. The Lymphoid Substance of the Chicken’s Harderian Gland is Organized in Two Histologically Distinct Compartments. Microsc Res Tech (1996) 34:166–76. doi: 10.1002/(SICI)1097-0029(19960601)34:2<166::AID-JEMT11>3.0.CO;2-O 8722712

[39] Deist MS Lamont SJ. What Makes the Harderian Gland Transcriptome Different From Other Chicken Immune Tissues? A Gene Expression Comparative Analysis. Front Physiol (2018) 0:492. doi: 10.3389/fphys.2018.00492 PMC5952037 29867543

[40] Murphy K Weaver C. Janeway’s Immunobiology. 9th edition. New York, NY: Garland Science/Taylor & Francis Group, LLC (2016).

[41] Cabeza-Cabrerizo M Cardoso A Minutti CM Pereira da Costa M Reis e Sousa C. Dendritic Cells Revisited. Annu Rev Immunol (2021) 39:131–66. doi: 10.1146/annurev-immunol-061020-053707 33481643

[42] Yun TJ Igarashi S Zhao H Perez OA Pereira MR Zorn E. Human Plasmacytoid Dendritic Cells Mount a Distinct Antiviral Response to Virus-Infected Cells. Sci Immunol (2021) 6:eabc7302. doi: 10.1126/sciimmunol.abc7302 33811059 PMC8221820

[43] Rehman ZU Umar S Meng C Ullah Z Riaz F Rehman SU. Dendritic Cell Harmonised Immunity to Poultry Pathogens; a Review. Worlds Poult Sci J (2017) 73:581–90. doi: 10.1017/S0043933917000496

[44] van den Biggelaar RHGA Arkesteijn GJA Rutten VPMG van Eden W Jansen CA. *In Vitro* Chicken Bone Marrow-Derived Dendritic Cells Comprise Subsets at Different States of Maturation. Front Immunol (2020) 11:141. doi: 10.3389/fimmu.2020.00141 32174908 PMC7054383

[45] Nagy N Bódi I Oláh I. Avian Dendritic Cells: Phenotype and Ontogeny in Lymphoid Organs. Dev Comp Immunol (2016) 58:47–59. doi: 10.1016/j.dci.2015.12.020 26751596

[46] Kaspers B Kaiser P. Avian Antigen-Presenting Cells. In: Avian Immunology. Munich, Germany: Elsevier (2014), 169–88. doi: 10.1016/B978-0-12-396965-1.00009-1

[47] Wu Z Kaiser P. Antigen Presenting Cells in a non-Mammalian Model System, the Chicken. Immunobiology (2011) 216:1177–83. doi: 10.1016/j.imbio.2011.05.012 21719145

[48] Vu Manh T-P Bertho N Hosmalin A Schwartz-Cornil I Dalod M. Investigating Evolutionary Conservation of Dendritic Cell Subset Identity and Functions. Front Immunol (2015) 6:260. doi: 10.3389/fimmu.2015.00260 26082777 PMC4451681

[49] Vu Manh T-P Marty H Sibille P Le Vern Y Kaspers B Dalod M. Existence of Conventional Dendritic Cells in Gallus Gallus Revealed by Comparative Gene Expression Profiling. J Immunol (2014) 192:4510–7. doi: 10.4049/jimmunol.1303405 24740508

[50] Sutton KM Morris KM Borowska D Sang H Kaiser P Balic A. Characterization of Conventional Dendritic Cells and Macrophages in the Spleen Using the CSF1R-Reporter Transgenic Chickens. Front Immunol (2021) 12:636436. doi: 10.3389/fimmu.2021.636436

[51] Liu G Yang H. Modulation of Macrophage Activation and Programming in Immunity. J Cell Physiol (2013) 228:502–12. doi: 10.1002/jcp.24157 22777800

[52] Prenen H Mazzone M. Tumor-Associated Macrophages: A Short Compendium. Cell Mol Life Sci (2019) 76:1447–58. doi: 10.1007/s00018-018-2997-3 PMC11105658 30747250

[53] Chaudhari AA Kim WH Lillehoj HS. Interleukin-4 (IL-4) may Regulate Alternative Activation of Macrophage-Like Cells in Chickens: A Sequential Study Using Novel and Specific Neutralizing Monoclonal Antibodies Against Chicken IL-4. Vet Immunol Immunopathol (2018) 205:72–82. doi: 10.1016/j.vetimm.2018.10.011 30459004

[54] Komohara Y Fujiwara Y Ohnishi K Takeya M. Tumor-Associated Macrophages: Potential Therapeutic Targets for Anti-Cancer Therapy. Adv Drug Delivery Rev (2016) 99:180–5. doi: 10.1016/j.addr.2015.11.009 26621196

[55] Cui L Ma Y Liang Y Zhang Y Chen Z Wang Z. Polarization of Avian Macrophages Upon Avian Flavivirus Infection. Vet Microbiol (2021) 256:109044. doi: 10.1016/j.vetmic.2021.109044 33836389

[56] Peng L van den Biggelaar RHGA Jansen CA Haagsman HP Veldhuizen EJA. A Method to Differentiate Chicken Monocytes Into Macrophages With Proinflammatory Properties. Immunobiology (2020) 225:152004. doi: 10.1016/j.imbio.2020.152004 33130516

[57] Rohde F Schusser B Hron T Farkašová H Plachý J Härtle S. Characterization of Chicken Tumor Necrosis Factor-α, a Long Missed Cytokine in Birds. Front Immunol (2018) 9:605. doi: 10.3389/fimmu.2018.00605 29719531 PMC5913325

[58] Tamura R Tanaka T Yamamoto Y Akasaki Y Sasaki H. Dual Role of Macrophage in Tumor Immunity. Immunotherapy (2018) 10:899–909. doi: 10.2217/imt-2018-0006 30073897

[59] Beug H von Kirchbach A Döderlein G Conscience J-F Graf T. Chicken Hematopoietic Cells Transformed by Seven Strains of Defective Avian Leukemia Viruses Display Three Distinct Phenotypes of Differentiation. Cell (1979) 18:375–90. doi: 10.1016/0092-8674(79)90057-6 227607

[60] Qureshi M. Avian Macrophage and Immune Response: An Overview. Poult Sci (2003) 82:691–8. doi: 10.1093/ps/82.5.691 PMC7194945 12762389

[61] Qureshi MA Miller L Lillehoj HS Ficken MD. Establishment and Characterization of a Chicken Mononuclear Cell Line. Vet Immunol Immunopathol (1990) 26:237–50. doi: 10.1016/0165-2427(90)90094-9 2176014

[62] Taylor PR Martinez-Pomares L Stacey M Lin H-H Brown GD Gordon S. Macrophage Receptors and Immune Recognition. Annu Rev Immunol (2005) 23:901–44. doi: 10.1146/annurev.immunol.23.021704.115816 15771589

[63] Sung Y-J Hotchkiss JH Austic RE Dietert RR. L-Arginine-Dependent Production of a Reactive Nitrogen Intermediate by Macrophages of a Uricotelic Species. J Leukoc Biol (1991) 50:49–56. doi: 10.1002/jlb.50.1.49 2056246

[64] Lin AW Chang CC McCormick CC. Molecular Cloning and Expression of an Avian Macrophage Nitric-Oxide Synthase cDNA and the Analysis of the Genomic 5′-Flanking Region. J Biol Chem (1996) 271:11911–9. doi: 10.1074/jbc.271.20.11911 8662618

[65] He H Kogut MH. CpG-ODN-Induced Nitric Oxide Production is Mediated Through Clathrin-Dependent Endocytosis, Endosomal Maturation, and Activation of PKC, MEK1/2 and P38 MAPK, and NF-κb Pathways in Avian Macrophage Cells (HD11). Cell Signal (2003) 15:911–7. doi: 10.1016/S0898-6568(03)00100-1 12873704

[66] Weining KC Schultz U Münster U Kaspers B Staeheli P. Biological Properties of Recombinant Chicken Interferon-γ. Eur J Immunol (1996) 26:2440–7. doi: 10.1002/eji.1830261026 8898958

[67] Breedveld A Kormelink TG van Egmond M de Jong EC. Granulocytes as Modulators of Dendritic Cell Function. J Leukoc Biol (2017) 102:1003–16. doi: 10.1189/jlb.4MR0217-048RR 28642280

[68] Khoyratty TE Ai Z Ballesteros I Eames HL Mathie S Martín-Salamanca S. Distinct Transcription Factor Networks Control Neutrophil-Driven Inflammation. Nat Immunol (2021) 22:1093–106. doi: 10.1038/s41590-021-00968-4 PMC7611586 34282331

[69] Ng LG Ostuni R Hidalgo A. Heterogeneity of Neutrophils. Nat Rev Immunol (2019) 19:255–65. doi: 10.1038/s41577-019-0141-8 30816340

[70] Kolaczkowska E Kubes P. Neutrophil Recruitment and Function in Health and Inflammation. Nat Rev Immunol (2013) 13:159–75. doi: 10.1038/nri3399 23435331

[71] Wen T Rothenberg ME. The Regulatory Function of Eosinophils. Microbiol Spectr (2016) 4:1–12. doi: 10.1128/microbiolspec.MCHD-0020-2015 PMC5088784 27780017

[72] Siracusa MC Kim BS Spergel JM Artis D. Basophils and Allergic Inflammation. J Allergy Clin Immunol (2013) 132:789–8. doi: 10.1016/j.jaci.2013.07.046 PMC3903395 24075190

[73] Juul-Madsen HR Viertlböeck B Härtle S Smith AL Göbel TW. Innate Immune Responses. In: Avian Immunology. Foulum, Denmark: Elsevier (2014). pp. 121–47. doi: 10.1016/B978-0-12-396965-1.00007-8

[74] Kaiser P. The Long View: A Bright Past, a Brighter Future? Forty Years of Chicken Immunology Pre- and Post-Genome. Avian Pathol (2012) 41:511–8. doi: 10.1080/03079457.2012.735359 23237363

[75] Genovese KJ He H Swaggerty CL Kogut MH. The Avian Heterophil. Dev Comp Immunol (2013) 41:334–40. doi: 10.1016/j.dci.2013.03.021 23583524

[76] Guriec N Bussy F Gouin C Mathiaud O Quero B Le Goff M. Ulvan Activates Chicken Heterophils and Monocytes Through Toll-Like Receptor 2 and Toll-Like Receptor 4. Front Immunol (2018) 9:2725. doi: 10.3389/fimmu.2018.02725 30532755 PMC6265352

[77] Kaiser P. Advances in Avian Immunology—Prospects for Disease Control: A Review. Avian Pathol (2010) 39:309–24. doi: 10.1080/03079457.2010.508777 20954007

[78] Kaiser P Stäheli P. Avian Cytokines and Chemokines. In: Avian Immunology. Edinburgh, Scotland, UK: Elsevier (2014), 189–204. doi: 10.1016/B978-0-12-396965-1.00010-8

[79] Maxwell MH. The Avian Eosinophil—A Review. Worlds Poult Sci J (1987) 43:190–207. doi: 10.1079/WPS19870013

[80] Marimuthu S Selvam R Kaninathan A D’Souza P. Effect of Dietary Supplementation of Phytogenic Feed Additive on Performance Traits, Serum Neopterin, and Cutaneous Basophil Hypersensitivity Response in Heat-Induced Stress Model of Broiler Chickens. J Adv Vet Anim Res (2020) 7:141–7. doi: 10.5455/javar.2020.g403 PMC7096127 32219120

[81] Maxwell MH Robertson GW. The Avian Basophilic Leukocyte: A Review. Worlds Poult Sci J (1995) 51:307–25. doi: 10.1079/WPS19950021

[82] Geiger TL Sun JC. Development and Maturation of Natural Killer Cells. Curr Opin Immunol (2016) 39:82–9. doi: 10.1016/j.coi.2016.01.007 PMC4801705 26845614

[83] Abel AM Yang C Thakar MS Malarkannan S. Natural Killer Cells: Development, Maturation, and Clinical Utilization. Front Immunol (2018) 9:1869. doi: 10.3389/fimmu.2018.01869 30150991 PMC6099181

[84] Pegram HJ Andrews DM Smyth MJ Darcy PK Kershaw MH. Activating and Inhibitory Receptors of Natural Killer Cells. Immunol Cell Biol (2011) 89:216–24. doi: 10.1038/icb.2010.78 20567250

[85] Chiang H-I Zhou H Raudsepp T Jesudhasan PR Zhu JJ. Chicken CD69 and CD94/NKG2-Like Genes in a Chromosomal Region Syntenic to Mammalian Natural Killer Gene Complex. Immunogenetics (2007) 59:603–11. doi: 10.1007/s00251-007-0220-z 17505822

[86] Meijerink N van Haarlem DA Velkers FC Stegeman AJ Rutten VPMG Jansen CA. Analysis of Chicken Intestinal Natural Killer Cells, a Major IEL Subset During Embryonic and Early Life. Dev Comp Immunol (2021) 114:103857. doi: 10.1016/j.dci.2020.103857 32891731

[87] Straub C Neulen M-L Sperling B Windau K Zechmann M Jansen CA. Chicken NK Cell Receptors. Dev Comp Immunol (2013) 41:324–33. doi: 10.1016/j.dci.2013.03.013 23542703

[88] Neulen M-L Viertlboeck BC Straub C Göbel TW. Identification of Novel Chicken CD4+ CD3– Blood Population With NK Cell Like Features. Dev Comp Immunol (2015) 49:72–8. doi: 10.1016/j.dci.2014.11.012 25445913

[89] Rogers SL Göbel TW Viertlboeck BC Milne S Beck S Kaufman J. Characterization of the Chicken C-Type Lectin-Like Receptors B-NK and B-Lec Suggests That the NK Complex and the MHC Share a Common Ancestral Region. J Immunol (2005) 174:3475–83. doi: 10.4049/jimmunol.174.6.3475 15749883

[90] Prager I Watzl C. Mechanisms of Natural Killer Cell-Mediated Cellular Cytotoxicity. J Leukoc Biol (2019) 105:1319–29. doi: 10.1002/JLB.MR0718-269R 31107565

[91] Wattrang E Magnusson SE Näslund K Thebo P Hagström Å Smith AL. Expression of Perforin, Granzyme A and Fas Ligand mRNA in Caecal Tissues Upon Eimeria Tenella Infection of Naïve and Immune Chickens. Parasite Immunol (2016) 38:419–30. doi: 10.1111/pim.12329 27136454

[92] Lo Nigro C Macagno M Sangiolo D Bertolaccini L Aglietta M Merlano MC. NK-Mediated Antibody-Dependent Cell-Mediated Cytotoxicity in Solid Tumors: Biological Evidence and Clinical Perspectives. Ann Transl Med (2019) 7:105. doi: 10.21037/atm.2019.01.42 31019955 PMC6462666

[93] Rogers SL Viertlboeck BC Göbel TW Kaufman J. Avian NK Activities, Cells and Receptors. Semin Immunol (2008) 20:353–60. doi: 10.1016/j.smim.2008.09.005 18948017

[94] Patel KR Roberts JT Barb AW. Multiple Variables at the Leukocyte Cell Surface Impact Fc γ Receptor-Dependent Mechanisms. Front Immunol (2019) 10:223. doi: 10.3389/fimmu.2019.00223 30837990 PMC6382684

[95] Viertlboeck BC Schweinsberg S Hanczaruk MA Schmitt R Du Pasquier L Herberg FW. The Chicken Leukocyte Receptor Complex Encodes a Primordial, Activating, High-Affinity IgY Fc Receptor. Proc Natl Acad Sci (2007) 104:11718–23. doi: 10.1073/pnas.0702011104 PMC1913898 17606923

[96] Fauriat C Long EO Ljunggren H-G Bryceson YT. Regulation of Human NK-Cell Cytokine and Chemokine Production by Target Cell Recognition. Blood (2010) 115:2167–76. doi: 10.1182/blood-2009-08-238469 PMC2844017 19965656

[97] He H Genovese KJ Kogut MH. Modulation of Chicken Macrophage Effector Function by TH1/TH2 Cytokines. Cytokine (2011) 53:363–9. doi: 10.1016/j.cyto.2010.12.009 21208811

[98] Göbel TW Kaspers B Stangassinger M. NK and T Cells Constitute Two Major, Functionally Distinct Intestinal Epithelial Lymphocyte Subsets in the Chicken. Int Immunol (2001) 13:757–62. doi: 10.1093/intimm/13.6.757 11369702

[99] Perera Molligoda Arachchige AS. Human NK Cells: From Development to Effector Functions. Innate Immun (2021) 27:212–29. doi: 10.1177/17534259211001512 PMC8054151 33761782

[100] Vervelde L Matthijs MGR van Haarlem DA de Wit JJ Jansen CA. Rapid NK-Cell Activation in Chicken After Infection With Infectious Bronchitis Virus M41. Vet Immunol Immunopathol (2013) 151:337–41. doi: 10.1016/j.vetimm.2012.11.012 PMC7112528 23245429

[101] Neulen M-L Göbel TW. Chicken CD56 Defines NK Cell Subsets in Embryonic Spleen and Lung. Dev Comp Immunol (2012) 38:410–5. doi: 10.1016/j.dci.2012.08.001 22922589

[102] Gunesch JT Dixon AL Ebrahim TA Berrien-Elliott MM Tatineni S Kumar T. CD56 Regulates Human NK Cell Cytotoxicity Through Pyk2. eLife (2020) 9:e57346. doi: 10.7554/eLife.57346 32510326 PMC7358009

[103] Mak TW Saunders ME Jett BD. NK, γδ T and NKT Cells. In: Primer to the Immune Response, 2nd ed. Boston: Academic Cell (2014), 247–68. doi: 10.1016/B978-0-12-385245-8.00011-X

[104] Paul S Lal G. Regulatory and Effector Functions of Gamma–Delta (γδ) T Cells and Their Therapeutic Potential in Adoptive Cellular Therapy for Cancer. Int J Cancer (2016) 139:976–85. doi: 10.1002/ijc.30109 27012367

[105] da Silva EZM Jamur MC Oliver C. Mast Cell Function: A New Vision of an Old Cell. J Histochem Cytochem (2014) 62:698–738. doi: 10.1369/0022155414545334 25062998 PMC4230976

[106] Galli SJ Gaudenzio N Tsai M. Mast Cells in Inflammation and Disease: Recent Progress and Ongoing Concerns. Annu Rev Immunol (2020) 38:49–77. doi: 10.1146/annurev-immunol-071719-094903 32340580

[107] Mukai K Tsai M Saito H Galli SJ. Mast Cells as Sources of Cytokines, Chemokines, and Growth Factors. Immunol Rev (2018) 282:121–50. doi: 10.1111/imr.12634 PMC5813811 29431212

[108] Varricchi G Raap U Rivellese F Marone G Gibbs BF. Human Mast Cells and Basophils—How are They Similar How are They Different? Immunol Rev (2018) 282:8–34. doi: 10.1111/imr.12627 29431214

[109] Baccari GC Pinelli C Santillo A Minucci S Rastogi RK. Mast Cells in Nonmammalian Vertebrates: An Overview. Int Rev Cell Mol Biol (2011) 290:1–53. doi: 10.1016/B978-0-12-386037-8.00006-5 21875561

[110] Ansari AR Arshad M Masood S Huang H-B Zhao X Li N. Salmonella Infection may Alter the Expression of Toll Like Receptor 4 and Immune Related Cells in Chicken Bursa of Fabricius. Microb Pathog (2018) 121:59–64. doi: 10.1016/j.micpath.2018.05.019 29763725

[111] Wang D Xiong J She R Liu L Zhang Y Luo D. Mast Cell Mediated Inflammatory Response in Chickens After Infection With Very Virulent Infectious Bursal Disease Virus. Vet Immunol Immunopathol (2008) 124:19–28. doi: 10.1016/j.vetimm.2008.01.005 18342956

[112] Machlus KR Italiano JE Jr. The Incredible Journey: From Megakaryocyte Development to Platelet Formation. J Cell Biol (2013) 201:785–96. doi: 10.1083/jcb.201304054 PMC3678154 23751492

[113] Helms CC Gladwin MT Kim-Shapiro DB. Erythrocytes and Vascular Function: Oxygen and Nitric Oxide. Front Physiol (2018) 9:125. doi: 10.3389/fphys.2018.00125 29520238 PMC5826969

[114] Ferdous F Scott TR. A Comparative Examination of Thrombocyte/Platelet Immunity. Immunol Lett (2015) 163:32–9. doi: 10.1016/j.imlet.2014.11.010 25448707

[115] Gaertner F Massberg S. Blood Coagulation in Immunothrombosis—At the Frontline of Intravascular Immunity. Semin Immunol (2016) 28:561–9. doi: 10.1016/j.smim.2016.10.010 27866916

[116] Ukidve A Zhao Z Fehnel A Krishnan V Pan DC Gao Y. Erythrocyte-Driven Immunization *via* Biomimicry of Their Natural Antigen-Presenting Function. Proc Natl Acad Sci USA (2020) 117:17727–36. doi: 10.1073/pnas.2002880117 PMC7395435 32665441

[117] Jones MP. Avian Hematology. Clin Lab Med (2015) 35:649–59. doi: 10.1016/j.cll.2015.05.013 26297411

[118] Ferdous F Saski C Bridges W Burns M Dunn H Elliott K. Transcriptome Profile of the Chicken Thrombocyte: New Implications as an Advanced Immune Effector Cell. PloS One (2016) 11:e0163890. doi: 10.1371/journal.pone.0163890 27711235 PMC5053482

[119] Ferdous F Scott T. Bacterial and Viral Induction of Chicken Thrombocyte Inflammatory Responses. Dev Comp Immunol (2015) 49:225–30. doi: 10.1016/j.dci.2014.11.019 25475960

[120] Paul MS Paolucci S Barjesteh N Wood RD Schat KA Sharif S. Characterization of Chicken Thrombocyte Responses to Toll-Like Receptor Ligands. PloS One (2012) 7:e43381. doi: 10.1371/journal.pone.0043381 22916253 PMC3423363

[121] Stier A Bize P Schull Q Zoll J Singh F Geny B. Avian Erythrocytes Have Functional Mitochondria, Opening Novel Perspectives for Birds as Animal Models in the Study of Ageing. Front Zool (2013) 10:33. doi: 10.1186/1742-9994-10-33 23758841 PMC3686644

[122] Morera D MacKenzie SA. Is There a Direct Role for Erythrocytes in the Immune Response? Vet Res (2011) 42:89. doi: 10.1186/1297-9716-42-89 21801407 PMC3199785

[123] Chen CH Göbel TWF Kubota T. Cooper MD. T Cell Development in the Chicken. Poult Sci (1994) 73:1012–8. doi: 10.3382/ps.0731012 7937462

[124] Smith AL Göbel TW. Avian T Cells. In: Avian Immunology. Oxford, England, UK: Elsevier (2014), 91–102. doi: 10.1016/B978-0-12-396965-1.00005-4

[125] Liu F Li J Lin IYC Yang X Ma J Chen Y. The Genome Resequencing of TCR Loci in Gallus Gallus Revealed Their Distinct Evolutionary Features in Avians. ImmunoHorizons (2020) 4:33–46. doi: 10.4049/immunohorizons.1900095 31992577

[126] Fenzl L Göbel TW Neulen M-L. γδ T Cells Represent a Major Spontaneously Cytotoxic Cell Population in the Chicken. Dev Comp Immunol (2017) 73:175–83. doi: 10.1016/j.dci.2017.03.028 28377199

[127] Morath A Schamel WW. αβ and γδ T Cell Receptors: Similar But Different. J Leukoc Biol (2020) 107:1045–55. doi: 10.1002/JLB.2MR1219-233R 31994778

[128] Ribot JC Lopes N Silva-Santos B. γδ T Cells in Tissue Physiology and Surveillance. Nat Rev Immunol (2021) 21:221–32. doi: 10.1038/s41577-020-00452-4 33057185

[129] Wiest DL. Development of γδ T Cells, the Special-Force Soldiers of the Immune System. In: Bosselut R Vacchio MS, editors. T-Cell Development Methods in Molecular Biology. New York, NY: Springer New York (2016). pp. 23–32. doi: 10.1007/978-1-4939-2809-5_2 26294395

[130] Mariuzza RA Agnihotri P Orban J. The Structural Basis of T-Cell Receptor (TCR) Activation: An Enduring Enigma. J Biol Chem (2020) 295:914–25. doi: 10.1074/jbc.REV119.009411 PMC6983839 31848223

[131] Berry R Headey SJ Call MJ McCluskey J Tregaskes CA Kaufman J. Structure of the Chicken CD3ϵδ/γ Heterodimer and its Assembly With the αβt Cell Receptor. J Biol Chem (2014) 289:8240–51. doi: 10.1074/jbc.M113.544965 PMC3961652 24488493

[132] Gouaillard C Huchenq-Champagne A Arnaud J Chen CH Rubin B. Evolution of T Cell Receptor (TCR) α β Heterodimer Assembly With the CD3 Complex. Eur J Immunol (2001) 31:3798–805. doi: 10.1002/1521-4141(200112)31:12<3798::AID-IMMU3798>3.0.CO;2-Z 11745401

[133] Alkie TN Yitbarek A Hodgins DC Kulkarni RR Taha-Abdelaziz K Sharif S. Development of Innate Immunity in Chicken Embryos and Newly Hatched Chicks: A Disease Control Perspective. Avian Pathol (2019) 48:288–310. doi: 10.1080/03079457.2019.1607966 31063007

[134] Parra D Takizawa F Sunyer JO. Evolution of B Cell Immunity. Annu Rev Anim Biosci (2013) 1:65–97. doi: 10.1146/annurev-animal-031412-103651 25340015 PMC4203447

[135] Glick B Chang TS Jaap RG. The Bursa of Fabricius and Antibody Production. Poult Sci (1956) 35:224–5. doi: 10.3382/ps.0350224

[136] Ratcliffe MJH Härtle S. B Cells, the Bursa of Fabricius and the Generation of Antibody Repertoires. In: Avian Immunology. Toronto, Canada: Elsevier (2014). pp. 65–89. doi: 10.1016/B978-0-12-396965-1.00004-2

[137] Wang J-Y. B Cells in Immunity and Tolerance. Singapore: Springer Singapore (2020). doi: 10.1007/978-981-15-3532-1

[138] Li A Rue M Zhou J Wang H Goldwasser MA Neuberg D. Utilization of Ig Heavy Chain Variable, Diversity, and Joining Gene Segments in Children With B-Lineage Acute Lymphoblastic Leukemia: Implications for the Mechanisms of VDJ Recombination and for Pathogenesis. Blood (2004) 103:4602–9. doi: 10.1182/blood-2003-11-3857 15010366

[139] Ko KH Lee IK Kim G Gu MJ Kim HY Park B-C. Changes in Bursal B Cells in Chicken During Embryonic Development and Early Life After Hatching. Sci Rep (2018) 8:16905. doi: 10.1038/s41598-018-34897-4 30442912 PMC6238004

[140] Nagy N Busalt F Halasy V Kohn M Schmieder S Fejszak N. In and Out of the Bursa—The Role of CXCR4 in Chicken B Cell Development. Front Immunol (2020) 11:1468. doi: 10.3389/fimmu.2020.01468 32765509 PMC7381227

[141] Lee L Samardzic K Wallach M Frumkin LR Mochly-Rosen D. Immunoglobulin Y for Potential Diagnostic and Therapeutic Applications in Infectious Diseases. Front Immunol (2021) 12:696003. doi: 10.3389/fimmu.2021.696003 34177963 PMC8220206

[142] Härtle S Magor KE Göbel TW Davison F Kaspers B. Structure and Evolution of Avian Immunoglobulins. In: Avian Immunology. Munich, Germany: Elsevier (2014). pp. 103–20. doi: 10.1016/B978-0-12-396965-1.00006-6

[143] Vadnais ML Criscitiello MF Smider VV. Antibodies From Other Species. In: Vaughan T Osbourn J Jallal B, editors. Methods and Principles in Medicinal Chemistry. Weinheim, Germany: Wiley-VCH Verlag GmbH & Co. KGaA (2017). pp. 85–112. doi: 10.1002/9783527699124.ch4

[144] Fitzgerald KA Kagan JC. Toll-Like Receptors and the Control of Immunity. Cell (2020) 180:1044–66. doi: 10.1016/j.cell.2020.02.041 PMC9358771 32164908

[145] Kogut MH Swaggerty C He H Pevzner I Kaiser P. Toll-Like Receptor Agonists Stimulate Differential Functional Activation and Cytokine and Chemokine Gene Expression in Heterophils Isolated From Chickens With Differential Innate Responses. Microbes Infect (2006) 8:1866–74. doi: 10.1016/j.micinf.2006.02.026 16815069

[146] Kaiser P Wu Z Rothwell L Fife M Gibson M Poh T-Y. Prospects for Understanding Immune-Endocrine Interactions in the Chicken. Gen Comp Endocrinol (2009) 163:83–91. doi: 10.1016/j.ygcen.2008.09.013 18957294

[147] Fulton JE McCarron AM Lund AR Pinegar KN Wolc A Chazara O. Miller MM. A High-Density SNP Panel Reveals Extensive Diversity, Frequent Recombination and Multiple Recombination Hotspots Within the Chicken Major Histocompatibility Complex B Region Between BG2 and CD1A1. Genet Sel Evol (2016) 48:1. doi: 10.1186/s12711-015-0181-x 26743767 PMC4705597

[148] Kaufman J. The Avian MHC. In: Avian Immunology. Cambridge, England, UK: Elsevier (2014), 149–67. doi: 10.1016/B978-0-12-396965-1.00008-X

[149] Aguado B Bahram S Beck S Campbell RD Forbes SA Geraghty D. The MHC Sequencing Consortium. Complete Sequence and Gene Map of a Human Major Histocompatibility Complex. Nature (1999) 401:921–3. doi: 10.1038/44853 10553908

[150] da Silva AP Gallardo RA. The Chicken MHC: Insights Into Genetic Resistance, Immunity, and Inflammation Following Infectious Bronchitis Virus Infections. Vaccines (2020) 8:637. doi: 10.3390/vaccines8040637 PMC7711580 33147703

[151] Bagchi S Yuan R Engleman EG. Immune Checkpoint Inhibitors for the Treatment of Cancer: Clinical Impact and Mechanisms of Response and Resistance. Annu Rev Pathol Mech Dis (2021) 16:223–49. doi: 10.1146/annurev-pathol-042020-042741 33197221

[152] Pardoll DM. The Blockade of Immune Checkpoints in Cancer Immunotherapy. Nat Rev Cancer (2012) 12:252–64. doi: 10.1038/nrc3239 PMC4856023 22437870

[153] Bernard D Hansen J Dupasquier L Lefranc M Benmansour A Boudinot P. Costimulatory Receptors in Jawed Vertebrates: Conserved CD28, Odd CTLA4 and Multiple BTLAs. Dev Comp Immunol (2007) 31:255–71. doi: 10.1016/j.dci.2006.06.003 16928399

[154] Kujundžić RN Lowenthal JW. The Role of Tryptophan Metabolism in iNOS Transcription and Nitric Oxide Production by Chicken Macrophage Cells Upon Treatment With Interferon Gamma. Immunol Lett (2008) 115:153–9. doi: 10.1016/j.imlet.2007.11.003 18082271

[155] Pereira MR Hang VR Vardiero E de Mello FG Paes-de-Carvalho R. Modulation of A1 Adenosine Receptor Expression by Cell Aggregation and Long-Term Activation of A2a Receptors in Cultures of Avian Retinal Cells: Involvement of the Cyclic AMP/PKA Pathway. J Neurochem (2010) 113:661–73. doi: 10.1111/j.1471-4159.2010.06641.x 20163523

[156] Reddy VRAP Mwangi W Sadigh Y Nair V. *In Vitro* Interactions of Chicken Programmed Cell Death 1 (PD-1) and PD-1 Ligand-1 (PD-L1). Front Cell Infect Microbiol (2019) 9:436. doi: 10.3389/fcimb.2019.00436 31921710 PMC6930881

[157] Selvaraj RK. Avian CD4+CD25+ Regulatory T Cells: Properties and Therapeutic Applications. Dev Comp Immunol (2013) 41:397–402. doi: 10.1016/j.dci.2013.04.018 23665004

[158] Borish LC Steinke JW. 2. Cytokines and Chemokines. J Allergy Clin Immunol (2003) 111:S460–75. doi: 10.1067/mai.2003.108 12592293

[159] Turner MD Nedjai B Hurst T Pennington DJ. Cytokines and Chemokines: At the Crossroads of Cell Signalling and Inflammatory Disease. Biochim Biophys Acta BBA - Mol Cell Res (2014) 1843:2563–82. doi: 10.1016/j.bbamcr.2014.05.014 24892271

[160] Kaiser P Poh TY Rothwell L Avery S Balu S Pathania US. A Genomic Analysis of Chicken Cytokines and Chemokines. J Interferon Cytokine Res (2005) 25:467–84. doi: 10.1089/jir.2005.25.467 16108730

[161] Gibson MS Kaiser P Fife M. The Chicken IL-1 Family: Evolution in the Context of the Studied Vertebrate Lineage. Immunogenetics (2014) 66:427–38. doi: 10.1007/s00251-014-0780-7 PMC4090809 24863340

[162] Giansanti F Giardi M Botti D. Avian Cytokines - An Overview. Curr Pharm Des (2006) 12:3083–99. doi: 10.2174/138161206777947542 16918436

[163] Medzhitov R. Origin and Physiological Roles of Inflammation. Nature (2008) 454:428–35. doi: 10.1038/nature07201 18650913

[164] Fleit HB. Chronic Inflammation. In: Pathobiology of Human Disease. Stony Brook, NY, USA: Elsevier (2014). pp. 300–14. doi: 10.1016/B978-0-12-386456-7.01808-6

[165] Hibino S Kawazoe T Kasahara H Itoh S Ishimoto T Sakata-Yanagimoto M. Inflammation-Induced Tumorigenesis and Metastasis. Int J Mol Sci (2021) 22:5421. doi: 10.3390/ijms22115421 34063828 PMC8196678

[166] Qu X Tang Y Hua S. Immunological Approaches Towards Cancer and Inflammation: A Cross Talk. Front Immunol (2018) 9:563. doi: 10.3389/fimmu.2018.00563 29662489 PMC5890100

[167] Chen L Deng H Cui H Fang J Zuo Z Deng J. Inflammatory Responses and Inflammation-Associated Diseases in Organs. Oncotarget (2017) 9:7204–18. doi: 10.18632/oncotarget.23208 PMC5805548 29467962

[168] Germolec DR Shipkowski KA Frawley RP Evans E. Markers of Inflammation. In: DeWitt JC Rockwell CE Bowman CC, editors. Immunotoxicity Testing Methods in Molecular Biology. New York, NY: Springer New York (2018), 57–79. doi: 10.1007/978-1-4939-8549-4_5 29882133

[169] Avery S Rothwell L Degen WDJ Schijns VEJC Young J Kaufman J. Characterization of the First Nonmammalian T2 Cytokine Gene Cluster: The Cluster Contains Functional Single-Copy Genes for IL-3, IL-4, IL-13, and GM-CSF, a Gene for IL-5 That Appears to be a Pseudogene, and a Gene Encoding Another Cytokinelike Transcript, KK34. J Interferon Cytokine Res Off J Int Soc Interferon Cytokine Res (2004) 24:600–10. doi: 10.1089/jir.2004.24.600 15626157

[170] Degen WGJ van Daal N van Zuilekom HI Burnside J Schijns VEJC. Identification and Molecular Cloning of Functional Chicken IL-12. J Immunol (2004) 172:4371–80. doi: 10.4049/jimmunol.172.7.4371 15034052

[171] Digby MR Lowenthal JW. Cloning and Expression of the Chicken Interferon-γ Gene. J Interferon Cytokine Res (1995) 15:939–45. doi: 10.1089/jir.1995.15.939 8590305

[172] Jakowlew SB Dillard PJ Kondaiah P Sporn MB Roberts AB. Complementary Deoxyribonucleic Acid Cloning of a Novel Transforming Growth Factor-β Messenger Ribonucleic Acid From Chick Embryo Chondrocytes. Mol Endocrinol (1988) 2:747–55. doi: 10.1210/mend-2-8-747 3211158

[173] Min W Lillehoj HS. Isolation and Characterization of Chicken Interleukin-17 cDNA. J Interferon Cytokine Res (2002) 22:1123–8. doi: 10.1089/10799900260442548 12513911

[174] Poh TY Pease J Young JR Bumstead N Kaiser P. Re-Evaluation of Chicken CXCR1 Determines the True Gene Structure: CXCLi1 (K60) AND CXCLi2 (CAF/INTERLEUKIN-8) ARE LIGANDS FOR THIS RECEPTOR*. J Biol Chem (2008) 283:16408–15. doi: 10.1074/jbc.M800998200 18417470

[175] Rothwell L Young JR Zoorob R Whittaker CA Hesketh P Archer A. Cloning and Characterization of Chicken IL-10 and Its Role in the Immune Response to Eimeria Maxima. J Immunol (2004) 173:2675–82. doi: 10.4049/jimmunol.173.4.2675 15294985

[176] Schneider K Klaas R Kaspers B Staeheli P. Chicken Interleukin-6. Eur J Biochem (2001) 268:4200–6. doi: 10.1046/j.1432-1327.2001.02334.x 11488913

[177] Rajakariar R Yaqoob MM Gilroy DW. COX-2 in Inflammation and Resolution. Mol Interv (2006) 6:199. doi: 10.1124/mi.6.4.6 16960142

[178] Nonaka M Kimura A. Genomic View of the Evolution of the Complement System. Immunogenetics (2006) 58:701–13. doi: 10.1007/s00251-006-0142-1 PMC2480602 16896831

[179] Lu Y-C Yeh W-C Ohashi PS. LPS/TLR4 Signal Transduction Pathway. Cytokine (2008) 42:145–51. doi: 10.1016/j.cyto.2008.01.006 18304834

[180] Poltorak A. Defective LPS Signaling in C3H/HeJ and C57BL/10ScCr Mice: Mutations in Tlr4 Gene. Science (1998) 282:2085–8. doi: 10.1126/science.282.5396.2085 9851930

[181] Harmon B. Avian Heterophils in Inflammation and Disease Resistance. Poult Sci (1998) 77:972–7. doi: 10.1093/ps/77.7.972 9657606

[182] Liu J Wang S Zhang Q Li X Xu S. Selenomethionine Alleviates LPS-Induced Chicken Myocardial Inflammation by Regulating the miR-128-3p-P38 MAPK Axis and Oxidative Stress. Metallomics (2020) 12:54–64. doi: 10.1039/c9mt00216b 31720660

[183] Zhang Y Guo F Ni Y Zhao R. LPS-Induced Inflammation in the Chicken is Associated With CCAAT/enhancer Binding Protein Beta-Mediated Fat Mass and Obesity Associated Gene Down-Regulation in the Liver But Not Hypothalamus. BMC Vet Res (2013) 9:257. doi: 10.1186/1746-6148-9-257 24345215 PMC3892065

[184] Zhao F Qu J Wang W Li S Xu S. The Imbalance of Th1/Th2 Triggers an Inflammatory Response in Chicken Spleens After Ammonia Exposure. Poult Sci (2020) 99:3817–22. doi: 10.1016/j.psj.2020.04.029 PMC7598003 32731967

[185] Khatri M Sharma JM. Response of Embryonic Chicken Lymphoid Cells to Infectious Bursal Disease Virus. Vet Immunol Immunopathol (2009) 127:316–24. doi: 10.1016/j.vetimm.2008.10.327 19081143

[186] Fellah JS Jaffredo T Nagy N Dunon D. Development of the Avian Immune System. In: Avian Immunology. Paris, France: Elsevier (2014), 45–63. doi: 10.1016/B978-0-12-396965-1.00003-0

[187] Hincke MT Da Silva MD Guyot N Gautron J McKee MD Guabiraba-Brito R. Dynamics of Structural Barriers and Innate Immune Components During Incubation of the Avian Egg: Critical Interplay Between Autonomous Embryonic Development and Maternal Anticipation. J Innate Immun (2019) 11:111–124. doi: 10.1159/000493719 30391943 PMC6738211

[188] Mast J Goddeeris BM. Development of Immunocompetence of Broiler Chickens. Vet Immunol Immunopathol (1999) 70:245–56. doi: 10.1016/S0165-2427(99)00079-3 10507364

[189] Ribatti D Tamma R Elieh Ali Komi D. The Morphological Basis of the Development of the Chick Embryo Immune System. Exp Cell Res (2019) 381:323–9. doi: 10.1016/j.yexcr.2019.05.027 31141709

[190] Guedes PT de Oliveira BCEPD de Abreu Manso PP Caputo LFG Cotta-Pereira G Pelajo-Machado M. Histological Analyses Demonstrate the Temporary Contribution of Yolk Sac, Liver, and Bone Marrow to Hematopoiesis During Chicken Development. PloS One (2014) 9:e90975. doi: 10.1371/journal.pone.0090975 24621665 PMC3951285

[191] Yvernogeau L Robin C. Restricted Intra-Embryonic Origin of Bona Fide Hematopoietic Stem Cells in the Chicken. Dev Camb Engl (2017) 144:2352–63. doi: 10.1242/dev.151613 PMC5536871 28526756

[192] Lowenthal JW Connick T McWaters PG York JJ. Development of T Cell Immune Responsiveness in the Chicken. Immunol Cell Biol (1994) 72:115–22. doi: 10.1038/icb.1994.18 8200687

[193] Marga Janse E Jeurissen SHM. Ontogeny and Function of Two Non-Lymphoid Cell Populations in the Chicken Embryo. Immunobiology (1991) 182:472–81. doi: 10.1016/S0171-2985(11)80211-1 1916887

[194] Pickel JM McCormack WT Chen C-IH Cooper MD Thompson CB. Differential Regulation of V(D)J Recombination During Development of Avian B and T Cells. Int Immunol (1993) 5:919–27. doi: 10.1093/intimm/5.8.919 8398986

[195] Six A Rast JP McCormack WT Dunon D Courtois D Li Y. Characterization of Avian T-Cell Receptor γ Genes. Proc Natl Acad Sci U.S.A. (1996) 93:15329–34. doi: 10.1073/pnas.93.26.15329 PMC26404 8986811

[196] Masteller EL. Thompson CB. B Cell Development in the Chicken. Poult Sci (1994) 73:998–1011. doi: 10.3382/ps.0730998 7937489

[197] Ratcliffe MJH Jacobsen KA. Rearrangement of Immunoglobulin Genes in Chicken B Cell Development. Semin Immunol (1994) 6:175–84. doi: 10.1006/smim.1994.1023 7948957

[198] Nuthalapati NK Evans JD Taylor RL Branton SL Nanduri B Pharr GT. Transcriptomic Analysis of Early B-Cell Development in the Chicken Embryo. Poult Sci (2019) 98:5342–54. doi: 10.3382/ps/pez354 PMC6771548 31237340

[199] Jansen CA van de Haar PM van Haarlem D van Kooten P de Wit S van Eden W. Identification of New Populations of Chicken Natural Killer (NK) Cells. Dev Comp Immunol (2010) 34:759–67. doi: 10.1016/j.dci.2010.02.009 20188123

[200] Dóra D Fejszák N Goldstein AM Minkó K Nagy N. Ontogeny of Ramified CD45 Cells in Chicken Embryo and Their Contribution to Bursal Secretory Dendritic Cells. Cell Tissue Res (2017) 368:353–70. doi: 10.1007/s00441-017-2595-y 28353134

[201] Balic A Garcia-Morales C Vervelde L Gilhooley H Sherman A Garceau V. Visualisation of Chicken Macrophages Using Transgenic Reporter Genes: Insights Into the Development of the Avian Macrophage Lineage. Development (2014) 141:3255–65. doi: 10.1242/dev.105593 PMC4197536 25063453

[202] Kannaki TR Reddy MR Verma PC Shanmugam M. Differential Toll-Like Receptor (TLR) mRNA Expression Patterns During Chicken Embryological Development. Anim Biotechnol (2015) 26:130–5. doi: 10.1080/10495398.2014.939658 25380465

[203] Kogut MH Holtzapple C Lowry VK Genovese K Stanker LH. Functional Responses of Neonatal Chicken and Turkey Heterophils Following Stimulation by Inflammatory Agonists. Am J Vet Res (1998) 59:1404–8. 9829397

[204] Kogut MH Lowry VK Moyes RB Bowden LL Bowden R Genovese K. Lymphokine-Augmented Activation of Avian Heterophils. Poult Sci (1998) 77:964–71. doi: 10.1093/ps/77.7.964 9657605

[205] Valdes TI Kreutzer D Moussy F. The Chick Chorioallantoic Membrane as a Novel *In Vivo* Model for the Testing of Biomaterials. J BioMed Mater Res (2002) 62:273–82. doi: 10.1002/jbm.10152 12209948

[206] Karaca T Yörük M Uslu S. Age-Related Changes in the Number of Mast Cells in the Avian Lymphoid Organs. Anat Histol Embryol (2006) 35:375–9. doi: 10.1111/j.1439-0264.2006.00698.x 17156090

[207] Lammers A Wieland WH Kruijt L Jansma A Straetemans T Schots A. Successive Immunoglobulin and Cytokine Expression in the Small Intestine of Juvenile Chicken. Dev Comp Immunol (2010) 34:1254–62. doi: 10.1016/j.dci.2010.07.001 20621117

[208] Anastasiadou M Michailidis G. Cytokine Activation During Embryonic Development and in Hen Ovary and Vagina During Reproductive Age and Salmonella Infection. Res Vet Sci (2016) 109:86–93. doi: 10.1016/j.rvsc.2016.09.016 27892879

[209] Abdul-Careem MF Hunter DB Lambourne MD Barta J Sharif S. Ontogeny of Cytokine Gene Expression in the Chicken Spleen. Poult Sci (2007) 86:1351–5. doi: 10.1093/ps/86.7.1351 17575181

[210] Beckers M Gladis-Villanueva M Hamann W Schmutzler W Zwadlo-Klarwasser G. The Use of the Chorio-Allantoic Membrane of the Chick Embryo as Test for Anti-Inflammatory Activity. Inflamm Res (1997) 46:29–30. doi: 10.1007/s000110050039 27517988

[211] Rosenbruch M Holst A. The Chick Embryo Yolk-Sac Blood Vessel System as an Experimental Model for Irritation and Inflammation. Toxicol In Vitro (1990) 4:327–31. doi: 10.1016/0887-2333(90)90075-5 20702189

[212] Zwadlo-Klarwasser G Görlitz K Hafemann B Klee D Klosterhalfen B. The Chorioallantoic Membrane of the Chick Embryo as a Simple Model for the Study of the Angiogenic and Inflammatory Response to Biomaterials. J Mater Sci Mater Med (2001) 12:195–9. doi: 10.1023/a:1008950713001 15348302

[213] Sharma BK Kakker NK Bhadouriya S Chhabra R. Effect of TLR Agonist on Infections Bronchitis Virus Replication and Cytokine Expression in Embryonated Chicken Eggs. Mol Immunol (2020) 120:52–60. doi: 10.1016/j.molimm.2020.02.001 32065987 PMC7112572

[214] Bar Shira E Friedman A. Innate Immune Functions of Avian Intestinal Epithelial Cells: Response to Bacterial Stimuli and Localization of Responding Cells in the Developing Avian Digestive Tract. PloS One (2018) 13:e0200393. doi: 10.1371/journal.pone.0200393 29979771 PMC6034880

[215] Ribatti D Tamma R Annese T. Chorioallantoic Membrane Vascularization. A Meta-Analysis. Exp Cell Res (2021) 405:112716. doi: 10.1016/j.yexcr.2021.112716 34186097

[216] Ribatti D. Advantages and Limitations of Chorioallantoic Membrane in Comparison With Other Classical In Vivo Angiogenesis Assays. In: Ribatti D, editor. The Chick Embryo Chorioallantoic Membrane in the Study of Angiogenesis and Metastasis: The CAM Assay in the Study of Angiogenesis and Metastasis. Dordrecht: Springer Netherlands (2010). pp. 75–85. doi: 10.1007/978-90-481-3845-6_7

[217] Burggren W Rojas Antich M. Angiogenesis in the Avian Embryo Chorioallantoic Membrane: A Perspective on Research Trends and a Case Study on Toxicant Vascular Effects. J Cardiovasc Dev Dis (2020) 7:56. doi: 10.3390/jcdd7040056 PMC7762154 33291457

[218] Ribatti D. Chorioallantoic Membrane in the Study of Angiogenesis, Antiangiogenesis, and the Vascularization of Grafted Tissues. In: Ribatti D, editor. The Chick Embryo Chorioallantoic Membrane in the Study of Angiogenesis and Metastasis: The CAM Assay in the Study of Angiogenesis and Metastasis. Dordrecht: Springer Netherlands (2010). pp. 17–40. doi: 10.1007/978-90-481-3845-6_2

[219] Kunz P Schenker A Sähr H Lehner B Fellenberg J. Optimization of the Chicken Chorioallantoic Membrane Assay as Reliable *In Vivo* Model for the Analysis of Osteosarcoma. PloS One (2019) 14:e0215312. doi: 10.1371/journal.pone.0215312 30986223 PMC6464229

[220] Deryugina EI. Chorioallantoic Membrane Microtumor Model to Study the Mechanisms of Tumor Angiogenesis, Vascular Permeability, and Tumor Cell Intravasation. In: Martin SG Hewett PW, editors. Angiogenesis Protocols Methods in Molecular Biology. New York, NY: Springer (2016). pp. 283–98. doi: 10.1007/978-1-4939-3628-1_19 27172961

[221] Mapanao AK Che PP Sarogni P Sminia P Giovannetti E Voliani V. Tumor Grafted – Chick Chorioallantoic Membrane as an Alternative Model for Biological Cancer Research and Conventional/Nanomaterial-Based Theranostics Evaluation. Expert Opin Drug Metab Toxicol (2021) 17:947–68. doi: 10.1080/17425255.2021.1879047 33565346

[222] Chu P-Y Koh AP-F Antony J Huang RY-J. Applications of the Chick Chorioallantoic Membrane as an Alternative Model for Cancer Studies. Cells Tissues Organs (2021), 1–16. doi: 10.1159/000513039 PMC9153341 33780951

[223] Stryker ZI Rajabi M Davis PJ Mousa SA. Evaluation of Angiogenesis Assays. Biomedicines (2019) 7:E37. doi: 10.3390/biomedicines7020037 31100863 PMC6631830

[224] Hillier LW Miller W Birney E Warren W Hardison RC Ponting CP. Sequence and Comparative Analysis of the Chicken Genome Provide Unique Perspectives on Vertebrate Evolution. Nature (2004) 432:695–716. doi: 10.1038/nature03154 15592404

[225] Rousset X Dosda E Viallet J. Use of an Egg Grafted With Tumor Cells in Order to Study the Anti-Cancer Effectiveness of Immune Therapies in the Absence of Immune Effector Cells Other Than Those in the Grafted Egg (2020). Available at: https://patentscope.wipo.int/search/en/detail.jsf?docId=WO2020089561 (Accessed September 16, 2021).

